# Doublet identification in single-cell sequencing data using scDblFinder

**DOI:** 10.12688/f1000research.73600.1

**Published:** 2021-09-28

**Authors:** Pierre-Luc Germain, Aaron Lun, Will Macnair, Mark D. Robinson

**Affiliations:** 1 DMLS Lab of Statistical Bioinformatics, University of Zürich, Zürich, 805, Switzerland; 2 D-HEST Institute for Neuroscience, ETH Zürich, Zürich, Switzerland; 3 Swiss Institute of Bioinformatics, University of Zürich, Zürich, Switzerland; 4 Genentech Inc., South San Francisco, CA, USA; 5 Pharma Research and Early Development, Neuroscience, Ophthalmology and Rare Diseases, F. Hoffmann-LaRoche Ltd, Basel, Switzerland

**Keywords:** single-cell sequencing, doublets, multiplets, filtering

## Abstract

Doublets are prevalent in single-cell sequencing data and can lead to artifactual findings. A number of strategies have therefore been proposed to detect them. Building on the strengths of existing approaches, we developed *scDblFinder*, a fast, flexible and accurate Bioconductor-based doublet detection method. Here we present the method, justify its design choices, demonstrate its performance on both single-cell RNA and accessibility sequencing data, and provide some observations on doublet formation, detection, and enrichment analysis. Even in complex datasets, *scDblFinder* can accurately identify most heterotypic doublets, and was already found by an independent benchmark to outcompete alternatives.

## Introduction

High-throughput single-cell sequencing, in particular single-cell/nucleus RNA-sequencing (scRNAseq), has provided an unprecedented resolution on biological phenomena. A particularly popular approach uses oil droplets or wells to isolate single cells along with barcoded beads. Depending on the cell density loaded, a proportion of reaction volumes (i.e. droplets or wells) will capture more than one cell, forming ‘doublets’ (or ‘multiplets’), i.e. two or more cells captured by a single reaction volume and thus sequenced as a single-cell artifact. The proportion of doublets has been shown to be proportional to the number of cells captured ( Bloom 2018; Kang *et al.* 2018). It is therefore at present common in single-cell experiments to have 10-20% doublets, making accurate doublet detection critical.

‘Homotypic’ doublets, which are formed by cells of the same type (i.e. similar transcriptional state), are very difficult to identify on the basis of their transcriptome alone ( McGinnis, Murrow, and Gartner 2019). They are also, however, relatively innocuous for most purposes, as they appear highly similar to singlets. ‘Heterotypic’ doublets (formed by cells of distinct transcriptional states), instead, can appear as an artifactual novel cell type and disrupt downstream analyses ( Germain, Sonrel, and Robinson 2020).

Experimental methods have been devised for detecting doublets in multiplexed samples, using barcodes ( Stoeckius *et al.* 2018) or genotypes (e.g. single-nucleotide polymorphisms) to identify droplets containing material from more than one sample ( Kang *et al.* 2018). While evidently useful, these identify only a fraction of the doublets, and fail to detect doublets formed by cells from the same sample, including heterotypic doublets.

A number of computational approaches have therefore been developed to identify doublets on the basis of their transcriptional profile ( McGinnis, Murrow, and Gartner 2019; DePasquale *et al.* 2019; Wolock, Lopez, and Klein 2019; Bais and Kostka 2020; Bernstein *et al.* 2020). Most of these approaches rely on the generation of artificial doublets by summing or averaging real cells, and score the similarity between them and the real cells. For example, DoubletFinder generates a *k*-nearest neighbor (kNN) graph on the union of real cells and artificial doublets, and estimates the density of artificial doublets in the neighborhood of each cell ( McGinnis, Murrow, and Gartner 2019). In a similar fashion, one of the methods proposed by Bais and Kostka (2020), bcds, generates artificial doublets and trains a classifier to distinguish them from real cells. Real cells that are classified with artificial doublets are then called as doublets. Finally, another strategy proposed by Bais and Kostka (2020) is a coexpression score, cxds, which flags cells that co-express a number of genes that otherwise tend to be mutually exclusive across cells.

Xi and Li (2021a) recently reported a benchmark of computational doublet detection methods, using both simulations and real datasets with genotype-based true doublets. Interestingly, despite several new publications, the benchmark identified the oldest method, *DoubletFinder* ( McGinnis, Murrow, and Gartner 2019), as the most accurate. However, another important observation from the benchmark was that no single method was systematically the best across all datasets, highlighting the necessity to test and benchmark methods across a variety of datasets, and suggesting that some strategies might have advantages and disadvantages across situations.

Here, we present the scDblFinder package, building on the extensive single-cell *Bioconductor* methods and infrastructures ( Amezquita *et al.* 2019) and implementing a number of doublet detection approaches. In particular, the *scDblFinder* method integrates insights from previous approaches and novel improvements to generate fast, flexible and robust doublet prediction. *scDblFinder* was independently tested by Xi and Li in the protocol extension to their initial benchmark and was found to have the best overall performance ( Xi and Li 2021b).

## Methods

### scDblFinder implementation

**Figure 1.**
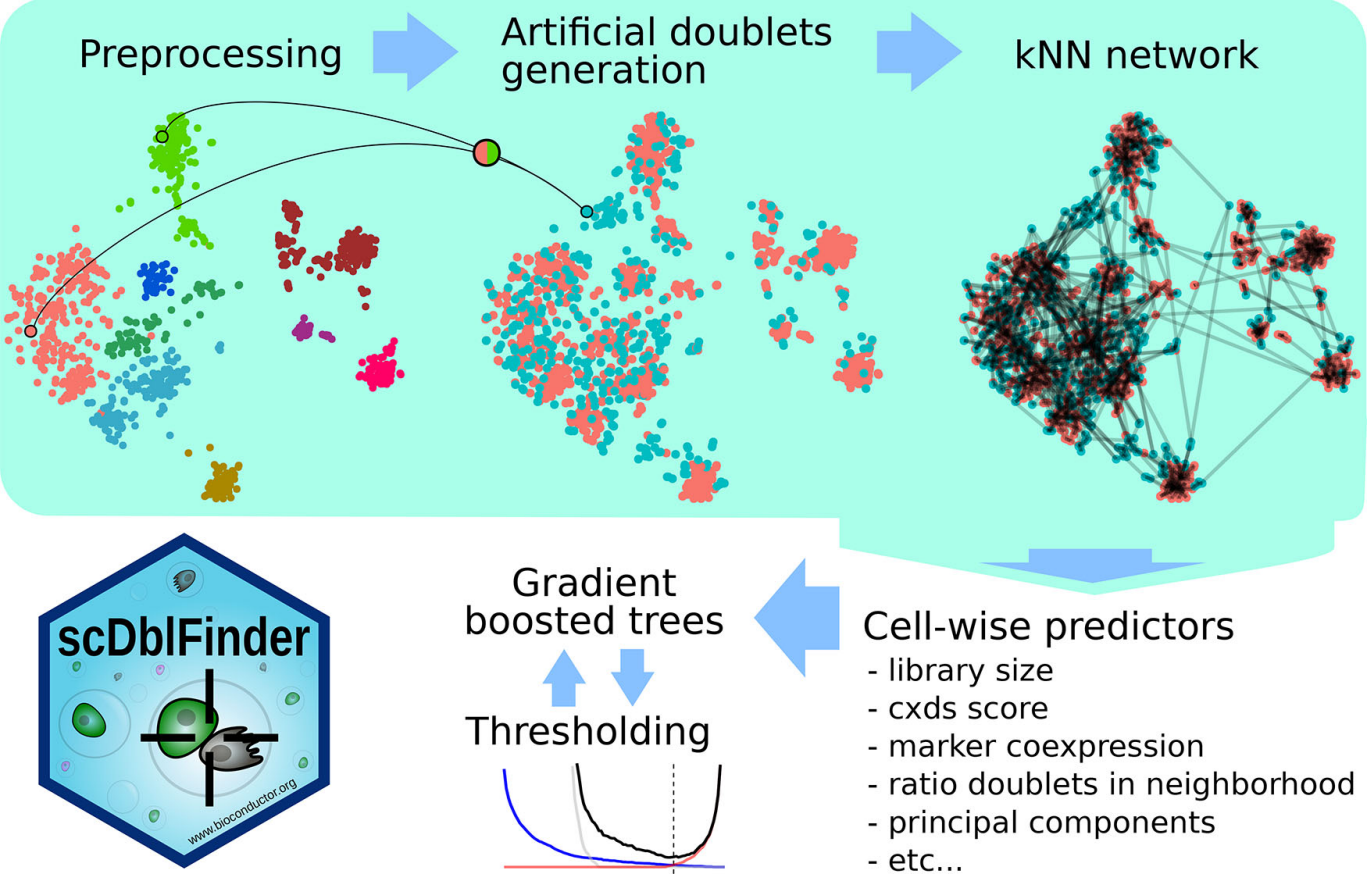
Overview of the scDblFinder method.

Figure 1 gives an overview of the *scDblFinder* method.

As a first step, the dataset is reduced to its top most expressed features (1000 by default); if the cluster-based approach is used, the top features per cluster are instead selected.

If using the cluster-based approach (and not manually specifying the clusters), a fast clustering is performed (see *Fast clustering*). Artificial doublets are then created by combining cells of different clusters, proportional to the cluster sizes. In explicitly concentrating on inter-cluster doublets, we do not attempt to identify homotypic doublets, which are virtually unidentifiable and relatively innocuous anyway. In doing so, we reduce the necessary number of artificial doublets (since no artificial doublet is ‘lost’ modeling homotypic doublets), and prevent the classifier from being trained to recognize cells that are indistinguishable from singlets (and would therefore call singlets as doublets). An alternative strategy, also available through *scDblFinder*, is to generate fully random artificial doublets, and use the iterative procedure (see below) to exclude unidentifiable artificial doublets from the training. In practice, the two approaches have comparable performances (see below), and they can also be combined.

Dimension reduction is then performed on the union of real cells and artificial doublets, and a nearest neighbor network is generated. The network is then used to estimate a number of characteristics for each cell, in particular the proportion of artificial doublets among the nearest neighbors. Rather than selecting a specific neighborhood size, the ratio is calculated at different values of *k*, creating multiple predictors that will be used by the classifier. A distance-weighted ratio is also included. Further cell-level predictors are added, including: projections on principal components; library size; and co-expression scores (based on a variation of Bais and Kostka 2020). *scDblFinder* then trains gradient boosted trees to distinguish, based on these features, artificial doublets from real cells. Finally, a thresholding procedure decides the score at which to call a cell by simultaneously minimizing the misclassification rate and the expected doublet rate (see Thresholding).

A key problem with classifier-based approaches is that some of the real cells are mislabeled, in the sense that they are in fact doublets labeled as singlets. These can mislead the classifier. For this reason, classification and thresholding are performed in an iterative fashion: at each round, the real cells identified as doublets are removed from the training data for the next round.

Using the benchmark datasets from Xi and Li (2021a), we next optimized a number of parameters in the procedure, notably regarding features to include and hyperparameters, so as to provide robust default parameters (see Germain 2021b, Figures 1-6). Some features, such as the distance to the nearest doublet or whether the nearest neighbor is an artificial doublet, had a negative impact on performance (see Germain 2021b, Figure 1), presumably because it led to over-fitting. Indeed, because artificial doublet creation can only approximate real doublets, a risk of classifier-based approaches is that the exact classification problem on which they are trained, namely distinguishing *artificial* doublets from *real* cells, slightly differs from the real problem on which they are expected to function (distinguishing *real* doublets from *real* singlets). To test the hypothesis that this can lead to overfitting, we used *scDblFinder* without the dimensional reduction and kNN steps, which arguably involve a loss of information, and trained the classifier directly on the expression of the selected genes. This resulted in a reduction in area under the precision and recall curve (AUPRC) in real datasets (see Germain 2021b, Figure 2; see also Figure 4). Finally, in line with a discrepancy between the trained and real problems, we observed that the variable importance calculated during training (see Germain 2021b, Figure 3) did not necessarily match that of the variable drop experiments (see Germain 2021b, Figure 1).

**Figure 2.**
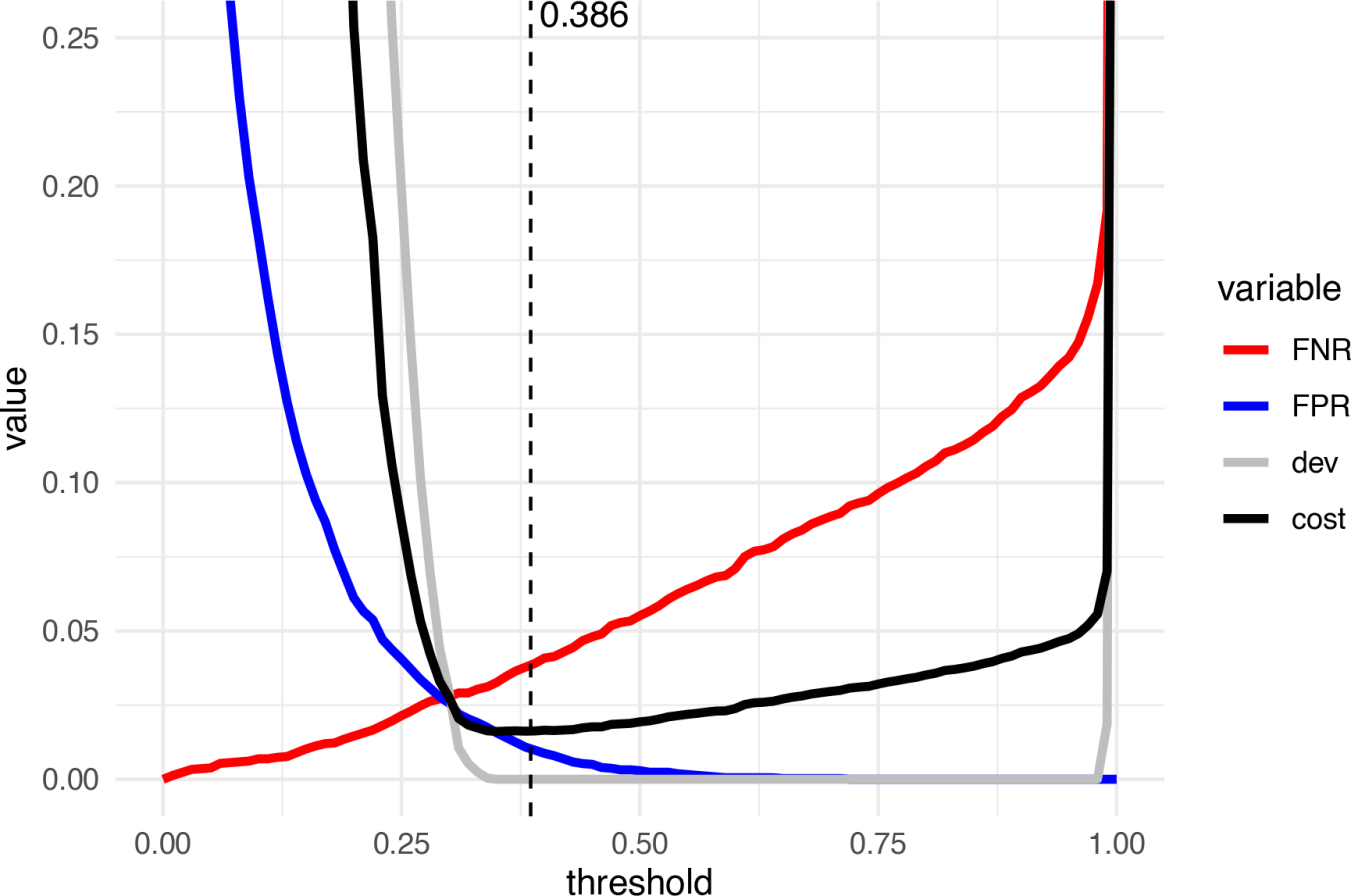
Illustration of the cost function to be minimized for thresholding. Plotted are the false negative rate (FNR; the rate of misclassified artificial doublets), the false positive rate (FPR; the proportion of real cells classified as doublets), the squared proportion deviation from the expected doublet rate (denoted ‘dev’), and the integrated cost function to be minimized (mean of the previous). The dashed line indicates the threshold called.

**Figure 3.**
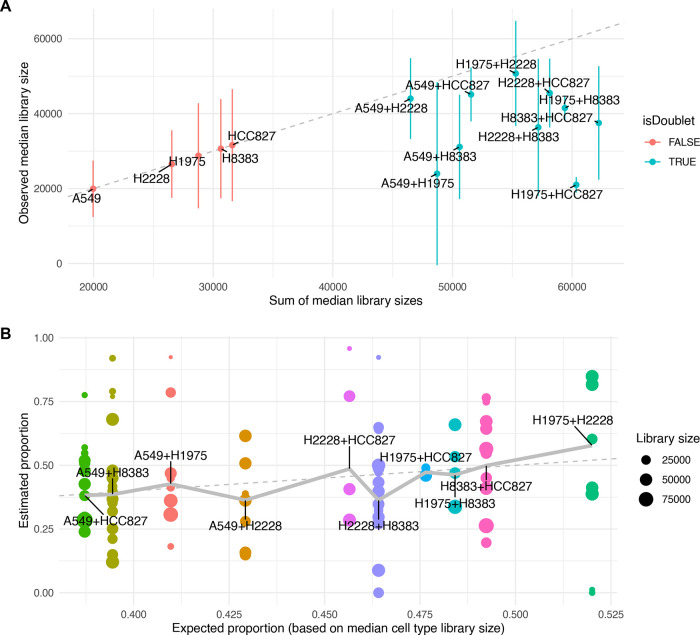
Characterization of real doublets. A: Observed median (and +/- one median absolute deviation in) library sizes per cell type against additive expectation for single cell and doublet types in a real dataset. The dashed line indicates the diagonal. B: Relative contribution of composing cell types in real doublets (each point represents a doublet) plotted against the expected relative contributions (based on the ratio between the median library sizes of the composing cell types). Values indicate the relative contribution of one of the two cell types to the doublet’s transcriptome. The dashed line indicates the diagonal, and the thick line indicates the weighted mean per doublet type.

We finally optimized hyperparameters (see Germain 2021b, Figure 4) as well as the number of iterations (see Germain 2021b, Figure 5), finding that a relatively low number of iterations (2-3) was sufficient.

**Figure 4.**
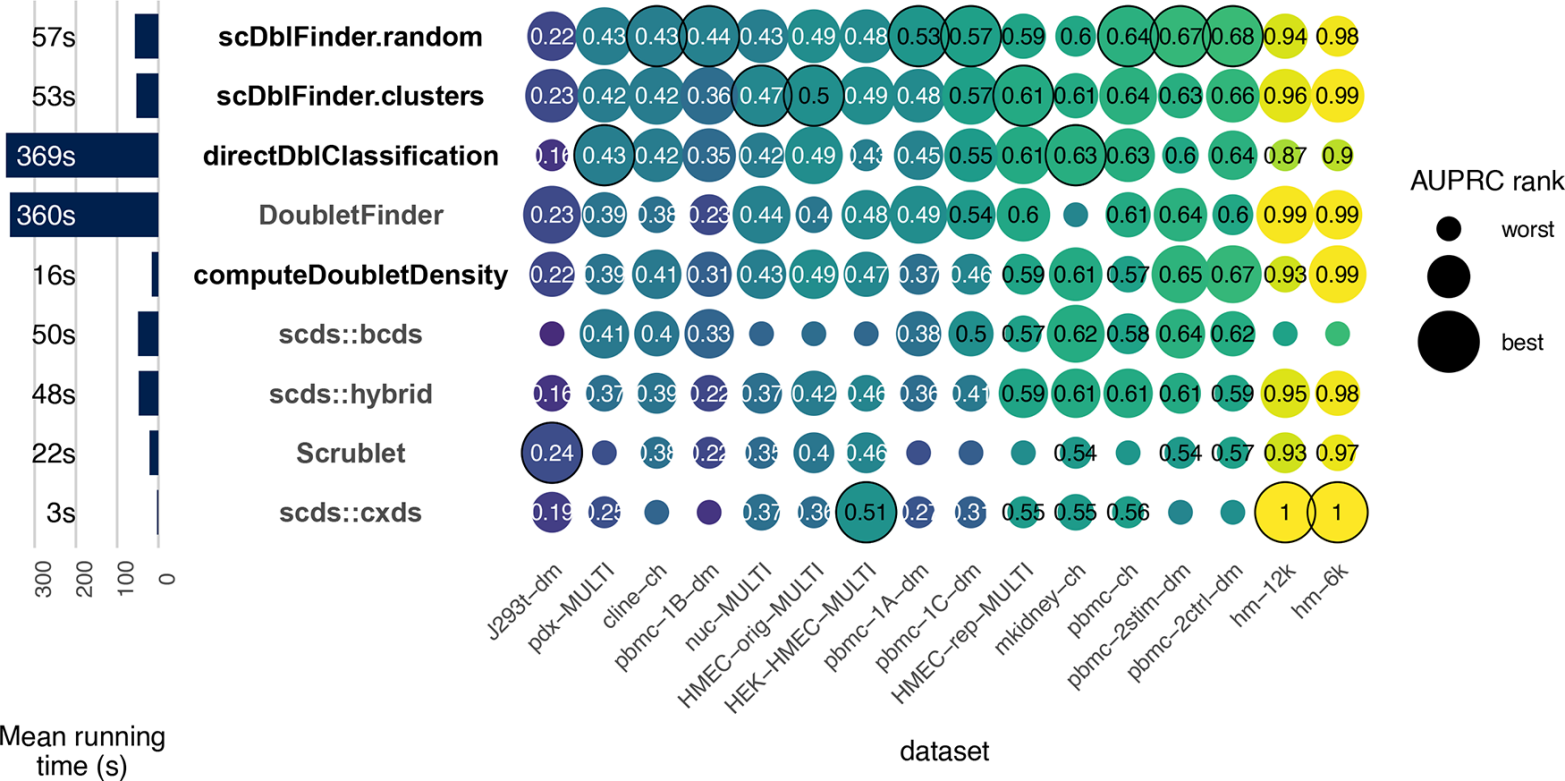
Benchmark. Accuracy (area under the precision and recall curve) of doublet identification using alternative methods across 16 benchmark datasets. The size of the dots indicate the relative ranking for the dataset, and the numbers indicate the actual area under the (PR) curve. For each dataset, the top method is circled in black. Methods in bold are available through the scDblFinder package.

**Figure 5.**
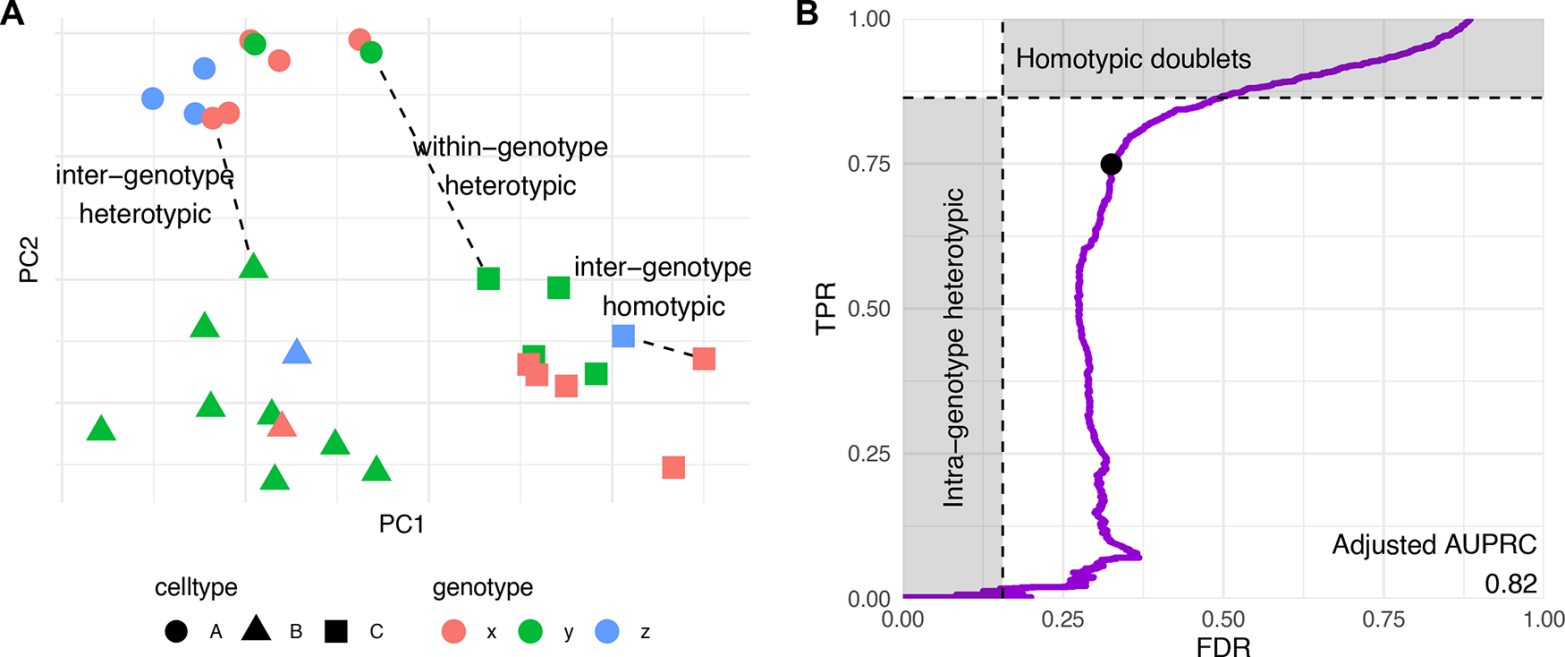
Doublet types and real accuracy of heterotypic doublet identification. A: Schematic (toy data) representing the different types of doublets. Within-genotype heterotypic doublets will wrongly be labeled as false positives, and inter-genotype homotypic will be labeled as false negatives. B: Adjusted PR curve for an example sample (GSM2560248). The two shaded areas represent the expected proportion of within-genotype heterotypic doublets (i.e. wrongly labeled as singlets in the truth) and inter-genotype homotypic doublets, respectively.

*Fast clustering*

Irlba-based singular value decomposition is first run using the scater package, and a kNN network is generated using the Annoy approximation implemented in BiocSingular. Louvain clustering is then used on the graph. If the dataset is sufficiently large (>1000 cells), a first rapid k-means clustering (using the mbkmeans package) is used to generate a large number of meta-cells, which are then clustered using the graph-based approach, propagating clusters back to the cells themselves.

*Thresholding*

Unless manually given, the expected number of doublets ( e ) is specified by $e=n^{2}/10^{-5}$ (where n is the number of cells captured). This is then restricted to heterotypic doublets using random expectation from cluster sizes or, if not using the cluster-based approach, using the proportion of artificial doublets misidentified. The doublet rate is accompanied by an uncertainty interval ( *dbr.sd* parameter), and the deviation from the expected doublet number for threshold t is then calculated as

$$\text{deviatio}n_{t}=\left\{\begin{array}{ll} 0 & \text{if}\left(o_{t},\geq ,e_{\mathrm{low}},\wedge ,o_{t},\leq ,e_{\mathrm{high}}\right)\\ 2\cdot \frac{\text{min}\left(\left|o_{t},-,e_{\mathrm{low}}\right|,,,\left|o_{t},-,e_{\mathrm{high}}\right|\right)}{e_{\mathrm{low}}+e_{\mathrm{high}}} & \text{otherwise} \end{array}\right.$$

where $o_{t}$ represents the number of real cells classified as doublets at threshold t , and $e_{\mathrm{low}}$ and $e_{\text{high}}$ represent, respectively, the lower and higher bounds of the expected number of heterotypic doublets in the dataset (based on the given or estimated doublet rate the *dbr.sd* parameter). The cost function being minimized is then simply given by ${\text{cost}}_{t}={\text{FNR}}_{t}+{\text{FPR}}_{t}+{\text{deviation}}_{t}^{2}$ , where the false negative rate ( ${\text{FNR}}_{t}$ ) represents the proportion of artificial doublets misclassified as singlets at threshold t , and the false positive rate ( ${\text{FPR}}_{t}$ ) represents the proportion of real cells classified as doublets. This is illustrated in Figure 2.

Since this is performed in an iterative fashion, the FPR is calculated ignoring cells which were called as doublets in the previous round.

### Doublet enrichment analysis

*Cluster stickiness*

Cluster ‘stickiness’ can be evaluated by fitting a single generalized linear model on the observed abundance of doublets of each origin, in the following way:

$$\log\left({\text{observed}}_{i}+0.1\right)=\log\left(e_{i}\right)+\beta_{z}\cdot \log\left({\text{difficulty}}_{i}\right)+\beta_{a}a_{i}+\beta_{b}b_{i}+\beta_{c}c_{i}+\ldots +\epsilon_{i},$$

where $\text{observe}d_{i}$ and $e_{i}$ represent the numbers of doublets formed by specific combination i of clusters which are respectively observed or expected from random combinations, and $a_{i}$ , $b_{i}$ and $c_{i}$ (etc) indicate whether or not (0/1) the doublet involves each cluster.

Because some doublets are easier to identify than others, some deviation from their expected abundance is typically observed. For this reason, a ${\text{difficulty}}_{i}$ term is optionally included, indicating the difficulty in identifying doublets of origin i , estimated from the misclassification of *scDblFinder*’s artificial doublets of that origin (by default, the term is included if at least seven clusters). A $\beta_{a}$ significantly different from zero, then, indicates that cluster *a* forms more or less doublets than expected – if positive, it indicates cluster ‘stickiness.’

For the (quasi-)binomial distributions, logit was used instead of log transformation, and the mean of observed and expected counts was used as observational weights.

*Enrichment for specific combinations*

To account for the different identification difficulty across doublet types, we first fit the following global negative binomial model:

$$\log\left(\text{observe}d_{i}\right)=\alpha +\log\left(e_{i}\right)+\beta \cdot \log\left(\text{difficult}y_{i}\right),$$

The fitted values are then considered the expected abundance, and the probability of each double type count given this expectation is calculated using either underlying distributions (for the negative binomial, the global over-dispersion parameter calculated in the first step is used).

### Direct classification

The direct classification approach is implemented in the *directDblClassification* function of the package. It uses the same doublet generation, thresholding and iterative learning procedures as *scDblFinder*, but trains directly on the normalized expression matrix of real and artificial cells instead of kNN-based features. The hyperparameters were the same except for the maximum tree depth, which was increased to six to account for the increased complexity of the predictors.

### Feature aggregation

For feature aggregation, *scDblFinder* first normalizes the counts using the Term Frequency - Inverse Document Frequency (TF-IDF) normalization, as implemented in Stuart *et al.* (2019). Principal component analysis (PCA) is then performed and the features are clustered into the desired number of meta-features using mini-batch k-means ( Hicks *et al.* 2021) or, if not available, simple k-means. The counts are then summed per meta-feature.

### scDblFinder operation

*scDblFinder* is provided as a bioconductor package. The input data for *scDblFinder* (denoted *x* below) can be either i) a count matrix (full or sparse), with genes/features as rows and cells/droplets as columns; or ii) an object of class SingleCellExperiment. In either case, the object should not contain empty drops, but should not otherwise have undergone very stringent filtering (which would bias the estimate of the doublet rate). The doublet detection can then be launched with:

*library (scDblFinder)*

*sce <- scDblFinder(x)*

The output is a SingleCellExperiment object including all of the input data, as well as a number of columns to the *colData* slot, the most important of which are:

• *sce$scDblFinder.score*: the final doublet score (the higher the more likely that the cell is a doublet)
• *sce$scDblFinder.class*: the binary classification (doublet or singlet)

scDblFinder can run on any system running R >= 4.0 and Bioconductor >= 3.12.

For more details, see the package’s vignettes.

## Results

### Characterization of real doublets

As most approaches rely on some comparison of real cells to artificial doublets, it is crucial to appropriately simulate doublets. To this end, we first characterized real doublets using a dataset of genetically distinct cell lines ( Tian *et al.* 2018). Because each cell line represents a distinct and more or less homogeneous transcriptional state, it is possible to identify the ‘cell types’ composing each doublet ( Figure 3). Although often larger, the median library sizes of doublets were systematically smaller than the sum of the median library sizes of composing cell types ( Figure 3A). We next investigated the relative contributions of the composing cell types using non-negative least square regression, expecting the larger cell types to contribute more to the doublet’s transcriptome.

Although differences in median library size across cell types were small (less than two-fold) compared to other datasets, we observed a weak association of the relative contributions with the relative sizes of the composing cell types ( Figure 3B, *p* = 2e-10). This effect was however considerably smaller than the variation within doublet type. This suggests that there are i) large variations in real cell size within a given cell type, and/or ii) large variations in the mRNA sampling efficiency that are independent for the two composing cells. In light of these ambiguities, we opted for a mixed strategy in the generation of artificial doublets: a proportion is generated by summing the libraries of individual cells, another by performing a poisson resampling of the obtained counts, and a third by re-weighting the contributions of cells depending on the relative median sizes of the composing cell types.

### scDblFinder outperforms alternative methods

A previous version of *scDblFinder* was already compared, and shown to be superior to existing alternatives in an independent benchmark by Xi and Li (2021a). Here we reproduced this benchmark using the most recent versions of the packages, and including variant methods from the *scDblFinder* package (among which the updated version of scran’s original method, and now available in the *scDblFinder* package as *computeDoubletDensity*). Figure 4 compares the performance of *scDblFinder* to alternatives across the real benchmark datasets. *scDblFinder* has the highest mean area under the precision-recall (PR) curve (see Germain 2021b, Figure 7), ranking first in a majority of datasets, and otherwise typically very close to the top. In addition, *scDblFinder* runs at a fraction of the time required by the next best methods ( Figure 4, left).

### Most heterotypic doublets are accurately identified

Several of the benchmark datasets have true doublets flagged by their mixing of single-nucleotide polymorphisms from multiple individuals ( Kang *et al.* 2018). In most of these cases, however, the doublets include also inter-individual homotypic doublets (in the sense of being a combination of cells of the same type from different individuals), which are difficult to detect from gene expression ( Figure 5A). In addition, they miss heterotypic doublets that are the result of the combination of different cell types from the same individual. Indeed, datasets where there is a full correspondence between cell type and individual (such as the human-mouse mixtures, e.g. hm-6k and hm-12k) typically have a much higher area under the Receiver-operator characteristic (ROC) and precision-recall (PR) curves ( Figure 4). It is therefore likely that the accuracy reported in the benchmark is below the actual one in detecting heterotypic doublets. Based on the frequency of the different individuals and cell types in a dataset, it is possible to infer the expected rate of inter-individual homotypic doublets and within-individual heterotypic doublets. This, in turns, allows us to adjust the measured true positive rate (TPR) and false discovery rate and get a better picture of our ability to detect heterotypic doublets. Figure 5B shows such an analysis for a complex dataset from Kang *et al.* (2018) . The inflection point of the PR curve roughly coincides with the expected proportion of heterotypic doublets among those flagged as true doublets.

Adjusting for both types of error in the truth, the area under the PR curve is considerably better (0.82 instead of 0.64), and at the automatic threshold we estimate that 87% of heterotypic doublets can be identified with a real FDR of 32% (a similar analysis for a different sample is shown in Germain 2021b, Figure 9).

### Flexible thresholding for doublet calling

**Figure 6.**
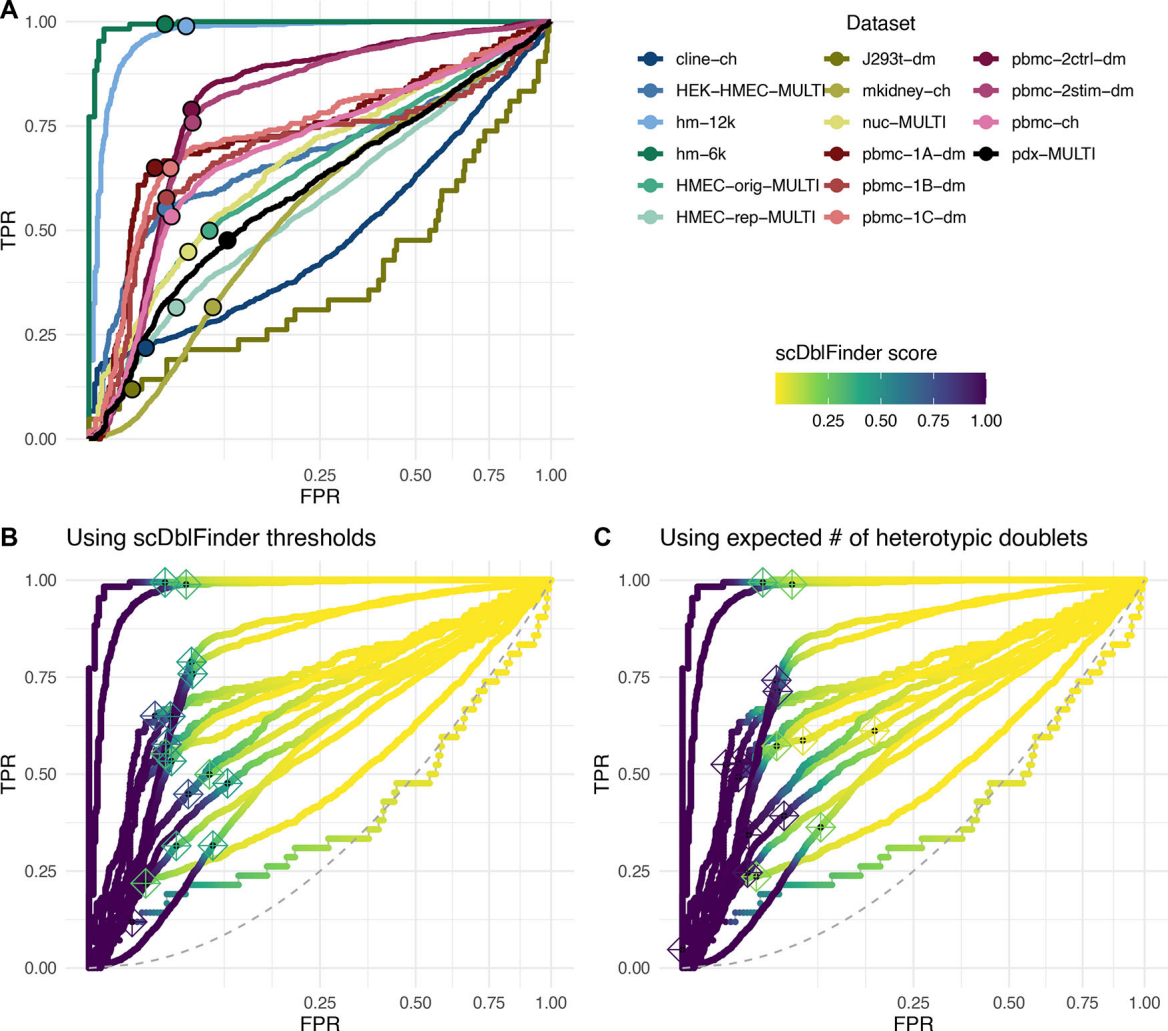
Thresholding. Receiver-operator characteristic (ROC) curves (with square-root transformation on the x axis) of the different benchmark datasets. In B-C, the colors indicate the scDblFinder doublet scores, and the crosses indicate the thresholds established through the thresholding method (B) or by taking the expected number of heterotypic doublets (C).

Most doublet detection methods provide a ‘doublet score’ that is higher in doublets than in singlets, and users are left to decide on a threshold above which cells will be excluded as doublets. Because *scDblFinder*’s scores come from a classifier, they can directly be interpreted as a probability. Nevertheless, a threshold needs to be set, and it should ideally be placed at the inflection point (assuming there is one) of the ROC or PR curve, so that most doublets and not too many singlets are excluded. While these curves are typically not available in practice, we found that in most cases the *scDblFinder* scores are rapidly changing from high to low very close to the inflection point ( Figure 6). One possibility is therefore to use directly a fixed probability threshold to call doublets. In some cases, however, there is a more gradual change in score (e.g. nuc-MULTI dataset), making it more difficult to establish a threshold in a non-arbitrary fashion. Building on the fairly tight relationship (especially in 10x-based datasets) between the number of cells captured and the rate of doublets generated ( Kang *et al.* 2018), another approach consists in setting the threshold based on the number of doublets (or heterotypic doublets) one expects to find in the data. *scDblFinder* includes a thresholding method that combines both rationales, and attempts to minimize both the proportion of artificial doublets being misclassified and the deviation from the expected doublet rate (see Thresholding).

The identified thresholds are shown in Figure 6A-B, and compared to thresholds based on the expected doublet rate in Figure 6C. In general, *scDblFinder* thresholds are closer to the inflection point.

### Doublet detection across multiple samples/captures

**Figure 7.**
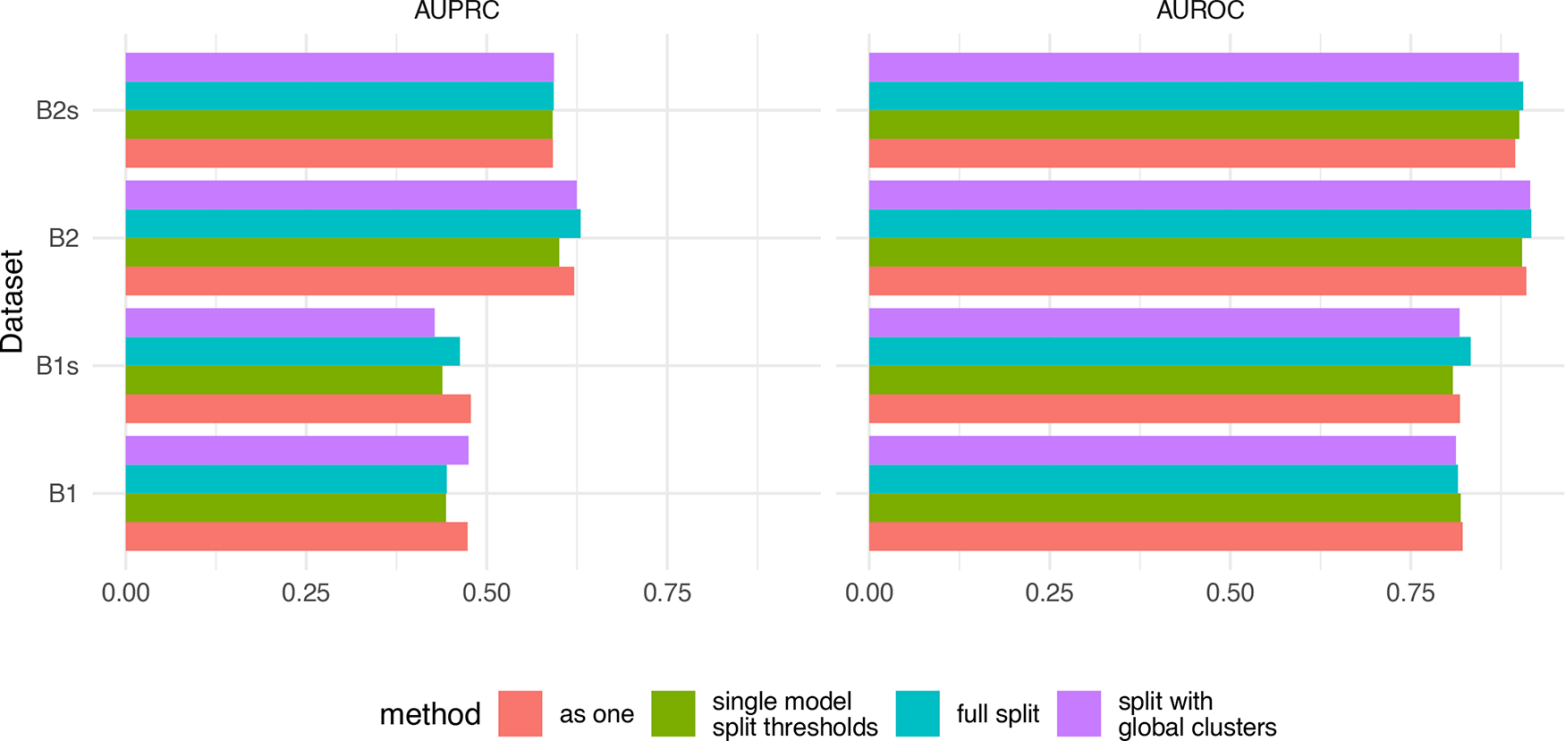
Comparison of four multi-sample strategies. B1 and B2 the two batches from dataset GSE96583, and contain 3 and 2 captures, respectively. The datasets with the suffix ‘s’ are versions downsampled to 30%. Using doublet detection on each capture separately (full split) was generally comparable to treating the captures as one (and adjusting the doublet rate).

Multiple samples are often profiled and analyzed together, with the very common risk of batch effects (either technical or biological) across samples ( Lütge *et al.* 2021). Therefore, while the cells from all samples might in principle provide more information for doublet detection than a single sample can afford on its own, this must be weighted against the risk of bias due to technical differences. To investigate this, we implemented different multi-sample approaches and tested them on two real multi-sample datasets with demuxlet-based true doublets, as well as a sub-sampling of them ( Figure 7).

The different multi-sample strategies had only a minor impact on the accuracy of the identification. Based on these results, the best overall strategy appears to be to process all samples as if they were one, however in our experience this can lead to biases against some samples when there are very large variations (e.g. in number of cells or coverage) across samples (not shown). This approach also greatly increases running time. In contrast, running the samples fully separately is computationally highly efficient, and is often equally accurate.

### scATACseq: aggregating rather than selecting features

**Figure 8.**
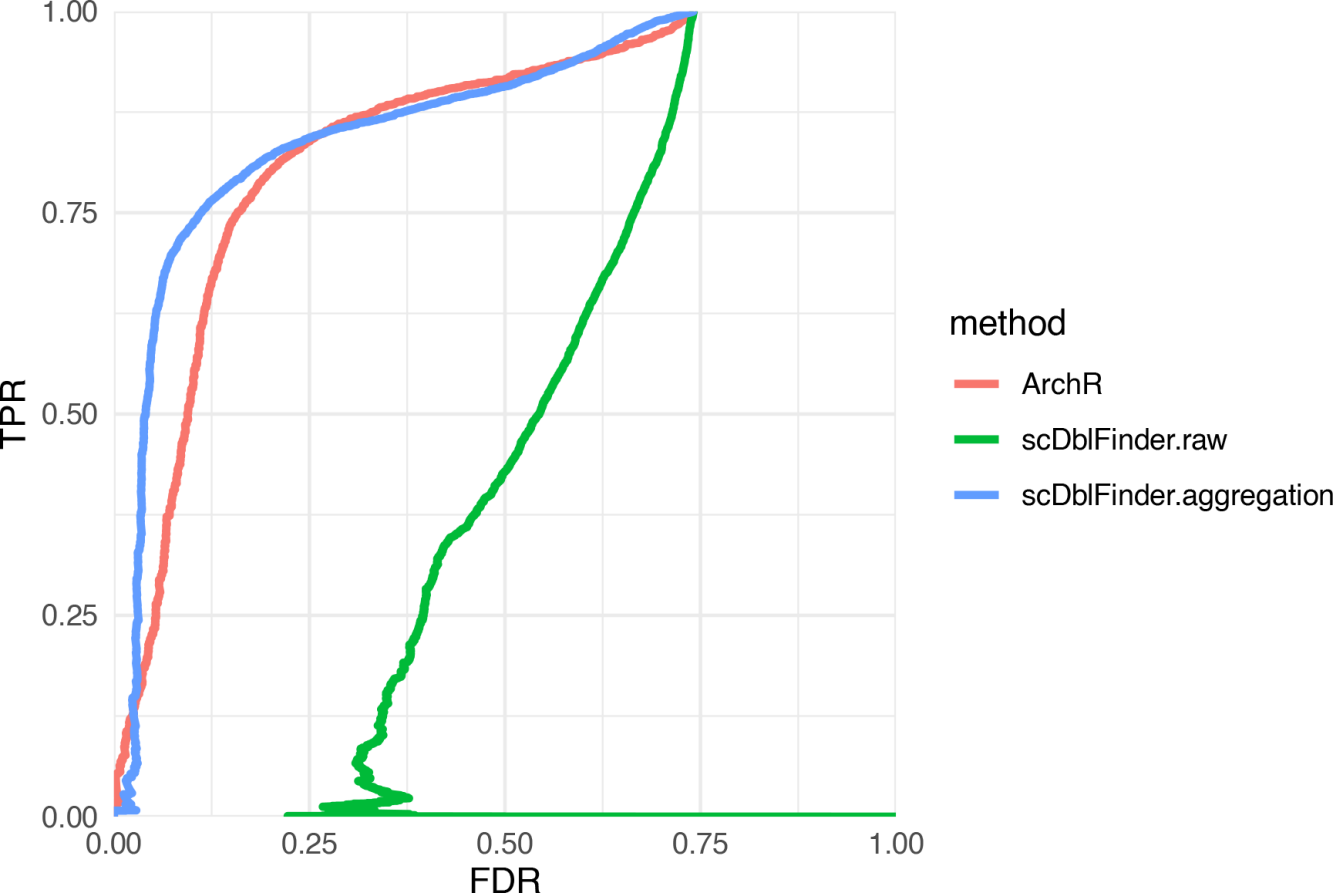
Doublet identification in single-nucleus ATAC-seq. Performance of *scDblFinder* with default (.raw) parameters or on aggregated features (.aggregation) versus ArchR (GSE162690 dataset).

We next investigated whether *scDblFinder* could be applied to other types of single-cell data prone to doublets, such as single-cell Assay for Transposase-Accessible Chromatin sequencing (ATACseq). To evaluate this, we used the mixture of 10 cell lines from Granja *et al.* (2021). With default parameters, *scDblFinder* performed very poorly ( Figure 8). This is chiefly because *scDblFinder* follows the common scRNAseq strategy of selecting an informative subset of the features, while ATACseq reads are typically sparsely distributed across the genome. However, working with all features (i.e. peaks) is computationally very expensive. An alternative to both approaches is to begin by reducing the size of the dataset by *aggregating* correlated features into a relatively small set, thereby using information from all. These aggregated features can then directly be used as the space in which to calculate distances. This method yielded equal or better performance than specialized single-cell ATACseq software ( Figure 8).

### Doublet origins and enrichment analysis

When artificial doublets are generated between clusters, we can keep track of the clusters composing them, and we reasoned that this information could be used to infer the clusters composing real doublets (hence after referred to as ‘doublet origin’). Using a simulation as well as the aforementioned real dataset with doublets of known origins (mixture of five cell lines from Tian *et al.* (2018)), we assessed the accuracy of doublet origin prediction based on the nearest artificial doublets in the kNN. These proved inaccurate, both in real and simulated data (see Germain 2021b, Figure 9A-B). Even training a classifier directly on this problem failed (see Germain 2021b, Figure 9C-D). The problem appears to be that, due to the very large variations in library sizes (and related variations in relative contributions of the composing cells – see Figure 3B), doublets often contain a large fraction of reads from one cell type, and conversely a small fraction from the other cell type. As a consequence, we can typically call at least one of the two originating cell types, but seldom both. In the real dataset, at least one of the two originating cell type is correctly identified in 73% of doublets (random expectation: 36%), but both are correct in only 20% of cases.

**Figure 9.**
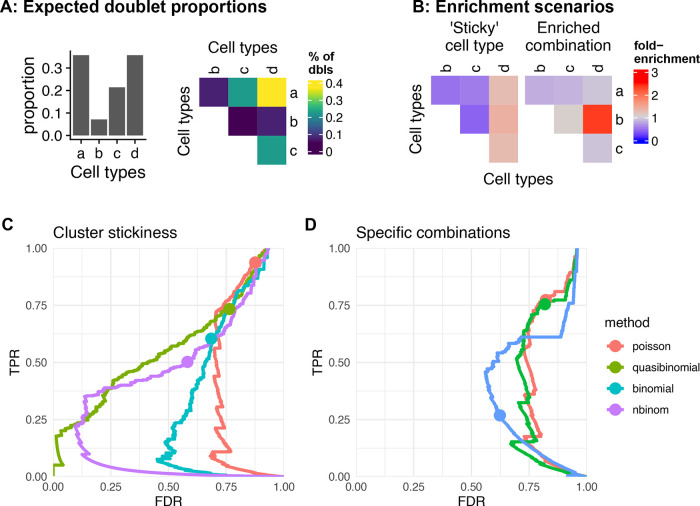
Doublet enrichment analysis. A, B: Doublet enrichment in a toy example. A: Proportion of different doublet types from random expectations based on the cell type abundances. B: The fold-enrichment over this expectation in two different doublet enrichment scenarios. C, D: Performance of the cluster stickiness tests (C) and tests for enrichment of specific combinations (D) using different underlying distributions.

While the identification of doublet origins remains a challenge, for the sake of completeness we nevertheless developed strategies to investigate whether certain doublet types were found more often than expected. Such enrichment could, for instance, indicate cell-to-cell interactions. We defined two forms of doublet enrichment ( Figure 9A-B), and specified models to test each possibility: i) enrichment in doublets formed by a specific combination of celltypes, or ii) enrichment in doublets involving a given cell type, denoted ‘sticky.’

The `stickiness’ of each cluster (as proxy for cell types) can be evaluated by fitting a single generalized linear model on the observed abundance of doublets of each origin (see Methods). We tested the performance of this test under different underlying distributions using simulated doublet counts. The number of doublets of each type is generated from random expectation with or without added stickiness (as factors of 1 to 3 on the probability) using negative binomial distributions with different over-dispersion parameters ( Figure 9C and Germain 2021b, Figure 10). The quasi-binomial showed the best performance, followed by the negative binomial, but in all cases the p-values were not well calibrated and many false positives were reported at a nominal FDR<0.05. This was robust across different over-dispersion values (see Germain 2021b, Figure 10).

We next sought to establish a test for the enrichment of specific combinations. Here, we simply computed the probability of the observed counts for each combination using different models (see Methods). We again tested this approach relying on different underlying distributions, on simulations with varying over-dispersion. The negative binomial performed best, however all variants suffered a high false discovery rate ( Figure 9C).

## Conclusions

The characterization of real doublets suggests a multi-layered variation in mRNA capture efficiency, and calls for a varied approach to artificial doublet generation. The *scDblFinder* package includes a set of efficient methods for doublet detection. In particular, the main *scDblFinder* approach uses mixed doublet generation approaches and integrates insights from previous approaches into a comprehensive doublet detection method that provides robustly accurate detection across a number of benchmark datasets, at a considerably greater speed and scalability than the best alternatives. Even in complex datasets, most heterotypic doublets can be accurately identified. Although the doublet scores given by *scDblFinder* can be directly interpreted as probabilities, simplifying their interpretation, the method also includes a trade-off thresholding procedure incorporating doublet rate expectations with classification optimization, thereby facilitating its usage. Finally, we further demonstrate that, with slight changes in parameters, the approach is also amenable to other data types such as single-nucleus ATAC-seq.

*scDblFinder* additionally provides utilities for identifying the origins of doublets (in terms of composing cell types) and testing for different forms of doublet enrichment. At present, however, the value of such tests is limited by the difficulty of accurately identifying doublet origins. Further research will be needed to assess to what extent this can be improved.

In conclusion, we believe that *scDblFinder*, with its flexibility, accuracy and scalability, represents a key resource for doublet detection in high-throughput single-cell sequencing data.

## Software availability

*scDblFinder* is available from Bioconductor: http://www.bioconductor.org/packages/release/bioc/html/scDblFinder.html.

The source code is available from: https://github.com/plger/scDblFinder.

Archived source code at time of publication: https://doi.org/10.6084/m9.figshare.16543518.v1 ( Germain, 2021a).

The software is released under the GNU Public License (GPL-3).

## Data availability

### Underlying data

figshare: scDblFinder. https://doi.org/10.6084/m9.figshare.16543518.v1 ( Germain, 2021a).

This repository contains the following underlying data:

• scDblFinder 1.7.4 (archived software version used in the paper).
• scDblFinder_paper (code to reproduce the analyses and figures).

The code to reproduce the analyses and figures is additionally available at https://github.com/plger/scDblFinder_paper.

Data are available under the terms of the Creative Commons Attribution 4.0 International license (CC-BY 4.0).

### Extended data

figshare: Supplementary Figures for the scDblFinder paper. https://doi.org/10.6084/m9.figshare.16617571.v1 ( Germain, 2021b)

This repository contains the following extended data:

• Supplementary Figures 1-10

Data are available under the terms of the Creative Commons Attribution 4.0 International license (CC-BY 4.0).
