# Doublet identification in single-cell sequencing data using *scDblFinder*

**DOI:** 10.12688/f1000research.73600.2

**Published:** 2022-05-16

**Authors:** Pierre-Luc Germain, Aaron Lun, Carlos Garcia Meixide, Will Macnair, Mark D. Robinson

**Affiliations:** 1 DMLS Lab of Statistical Bioinformatics, University of Zürich, Zürich, 805, Switzerland; 2 D-HEST Institute for Neuroscience, ETH Zürich, Zürich, Switzerland; 3 Swiss Institute of Bioinformatics, University of Zürich, Zürich, Switzerland; 4 Genentech Inc., South San Francisco, CA, USA; 5 Pharma Research and Early Development, Neuroscience, Ophthalmology and Rare Diseases, F. Hoffmann-LaRoche Ltd, Basel, Switzerland

**Keywords:** single-cell sequencing, doublets, multiplets, filtering

## Abstract

Doublets are prevalent in single-cell sequencing data and can lead to artifactual findings. A number of strategies have therefore been proposed to detect them. Building on the strengths of existing

approaches, we developed *scDblFinder*, a fast, flexible and accurate Bioconductor-based doublet detection method. Here we present the method, justify its design choices, demonstrate its performance on both single-cell RNA and accessibility (ATAC) sequencing data, and provide some observations on doublet formation, detection, and enrichment analysis. Even in complex datasets, *scDblFinder* can accurately identify most heterotypic doublets, and was already found by an independent benchmark to outcompete alternatives.

## Introduction

High-throughput single-cell sequencing, in particular single-cell/nucleus RNA-sequencing (scRNAseq), has provided an unprecedented resolution on biological phenomena. A particularly popular approach uses oil droplets or wells to isolate single cells along with barcoded beads. Depending on the cell density loaded, a proportion of reaction volumes (i.e. droplets or wells) will capture more than one cell, forming ‘doublets’ (or ‘multiplets’), i.e. two or more cells captured by a single reaction volume and thus sequenced as a single-cell artifact. The proportion of doublets has been shown to be proportional to the number of cells captured ( Bloom 2018; Kang *et al.* 2018). It is therefore at present common in single-cell experiments to have 10-20% doublets, making accurate doublet detection critical.

To avoid confusion, we will denote as ‘droplet’ the reads that are assigned to one barcode (either doublet or singlet), and reserve the term ‘cells’ to talk about original (singlet) cells. ‘Homotypic’ doublets, which are formed by cells of the same type (i.e. similar transcriptional state), are very difficult to identify on the basis of their transcriptome alone ( McGinnis, Murrow, and Gartner 2019). They are also, however, relatively innocuous for most purposes, as they appear highly similar to singlets. ‘Heterotypic’ doublets (formed by cells of distinct transcriptional states), instead, can appear as an artifactual novel cell type and disrupt downstream analyses ( Germain, Sonrel, and Robinson 2020).

Experimental methods have been devised for detecting doublets in multiplexed samples, using barcodes ( Stoeckius *et al.* 2018) or genotypes (e.g. single-nucleotide polymorphisms) to identify droplets containing material from more than one sample ( Kang *et al.* 2018). While evidently useful, these often incur additional costs or limitations. Furthermore, they identify only a fraction of the doublets, and fail to detect doublets formed by cells from the same sample, including heterotypic doublets. The proportion of doublets missed will decrease with the degree of multiplexing, but even mixing 10 samples would result in 10% of the doublets missed; moreover, these approaches are not always applicable.

A number of computational approaches have therefore been developed to identify doublets on the basis of their transcriptional profile ( McGinnis, Murrow, and Gartner 2019; DePasquale *et al.* 2019; Wolock, Lopez, and Klein 2019; Bais and Kostka 2020; Bernstein *et al.* 2020). Most of these approaches rely on the generation of artificial doublets by summing or averaging reads from real droplets, and score the similarity between them and the real droplets. For example, DoubletFinder generates a *k*-nearest neighbor (kNN) graph on the union of real droplets and artificial doublets, and estimates the density of artificial doublets in the neighborhood of each droplet ( McGinnis, Murrow, and Gartner 2019). In a similar fashion, one of the methods proposed by Bais and Kostka (2020), *bcds*, generates artificial doublets and trains a classifier to distinguish them from real cells. Real cells that are classified with artificial doublets are then called as doublets. Finally, another strategy proposed by Bais and Kostka (2020) is a coexpression score, *cxds*, which flags droplets that co-express a number of genes that otherwise tend to be mutually exclusive across droplets.

Xi and Li (2021a) recently reported a benchmark of computational doublet detection methods, using both simulations and real datasets with annotated true doublets. Interestingly, despite several new publications, the initial benchmark found the oldest method, *DoubletFinder* ( McGinnis, Murrow, and Gartner 2019), to outperform others. However, another important observation from the benchmark was that no single method was systematically the best across all datasets, highlighting the necessity to test and benchmark methods across a variety of datasets, and suggesting that some strategies might have advantages and disadvantages across situations.

Here, we present the scDblFinder package, building on the extensive single-cell *Bioconductor* methods and infrastructures ( Amezquita *et al.* 2019) and implementing a number of doublet detection approaches. In particular, the *scDblFinder* method integrates insights from previous approaches and novel improvements to generate fast, flexible and robust doublet prediction. *scDblFinder* was independently tested by Xi and Li in the protocol extension to their initial benchmark and was found to have the best overall performance ( Xi and Li 2021b).

## Results

### Simulation of artificial doublets

**Figure 1.**
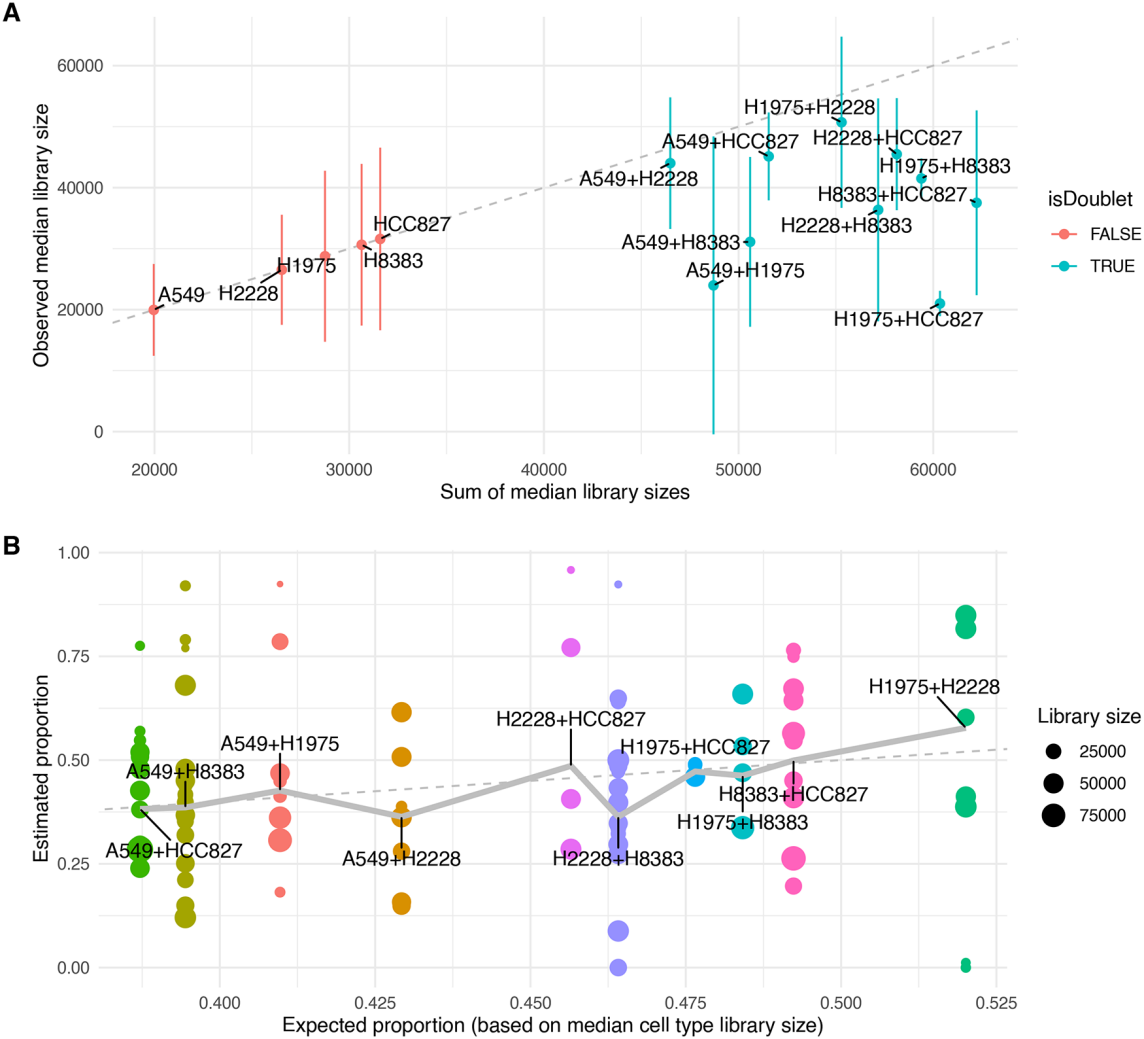
Characterization of real doublets in a mixture of three human lung adenocarcinoma cell lines. A: Observed median (and +/- one median absolute deviation in) library sizes per cell type against additive expectation for single cell and doublet types in a real dataset. The dashed line indicates the diagonal. B: Relative contribution of composing cell types in real doublets (each point represents a doublet) plotted against the expected relative contributions (based on the ratio between the median library sizes of the composing cell types). Values indicate the relative contribution of one of the two cell types to the doublet’s transcriptome. The dashed line indicates the diagonal, and the thick line indicates the weighted mean per doublet type. The annotation of cell types and their combinations comes from the original Demuxlet analysis by Tian et al., excluding ambiguous calls.

As most approaches rely on some comparison of real droplets to artificial doublets, it is crucial to appropriately simulate doublets. To this end, we first characterized real doublets using a dataset of genetically distinct cell lines ( Tian *et al.* 2018). Because each cell line represents a distinct and more or less homogeneous transcriptional state, it is possible, using genotypes, to identify the ‘cell types’ composing each doublet ( Figure 1). Although often larger, the median library sizes of doublets were systematically smaller than the sum of the median library sizes of composing cell types ( Figure 1A).

We next investigated the relative contributions of the composing cell types using non-negative least square regression, expecting the larger cell types to contribute more to the doublet’s transcriptome.

Although differences in median library size across cell types were small (less than two-fold) compared to other datasets, we observed an association of the relative contributions with the relative sizes of the composing cell types ( Figure 1B, *p* = 2e-10). However, this effect was very weak - considerably smaller than the variation within doublet type. This suggests that there are i) large variations in real cell size within a given cell type, and/or ii) large variations in the mRNA sampling efficiency that are independent for the two composing cells. In any case, for many doublets, the two composing cell types contribute very unequally. This also explains why, while doublets sometimes form their own clusters, they often appear at the periphery of the singlet cluster they most resemble.

In light of these ambiguities, we opted for a mixed strategy to simulate artificial doublets, generating them in a combination of three different ways (see Methods): a proportion is generated by summing the libraries of individual droplets, another by performing a Poisson resampling of the obtained counts, and a third by re-weighting the contributions of cells depending on the relative median sizes of the composing cell types (in case the observed library size is a poor indicator of RNA content). This strategy did not lead to a clear overall improvement across the datasets ( *Extended data* – Figure 1A) over the simple sum (both of which were clearly superior to averaging), suggesting that most of the difference is anyway within the wide variability in library sizes, and/or that the normalization and dimensionality reduction steps are sufficient to remove remaining differences between real and artificial doublets. Another possible interpretation is that doublets can be approximated as the sum of the counts of the composing cells, but that doublets composed of larger cells are less likely to form. Either way, since it was not deleterious and might prove more robust to variations in protocols, we nevertheless maintained the mixed strategy, generating the majority of doublets (75%) using the simple sum, and the rest using the mixed strategy.

### scDblFinder consistently outperforms alternative methods

**Figure 2.**
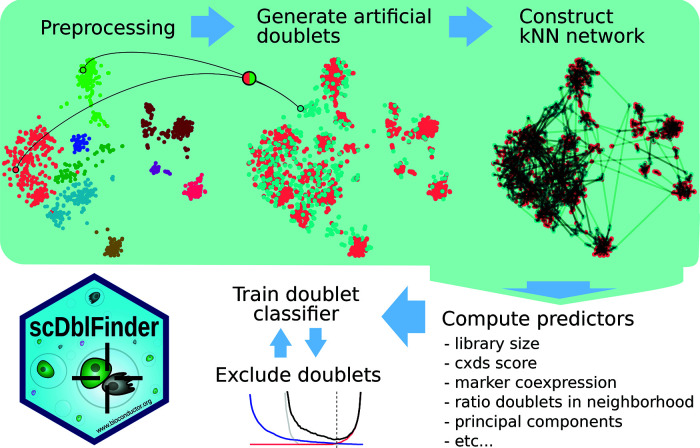
Overview of the *scDblFinder* method.

Figure 2 gives an overview of the *scDblFinder* method (see Methods for details). Briefly, after some initial processing, artificial doublets (either random or between-cluster, depending on the settings) are generated, then a nearest neighbor (kNN) network is generated. Rather than selecting a single neighborhood size, as most kNN-based methods do, *scDblFinder* gathers statistics at various neighborhood sizes, thereby enabling the downstream classifier to select the most informative size(s), which might also differ across the expression space. Various characteristics from each cell/doublet and its neighborhood (such as the density of artificial doublets in the neighborhood) are then gathered to build a cell-level predictors matrix. On the basis of these predictors, a classifier is trained to distinguish artificial doublets from droplets. A key problem with classifier-based approaches is that some of the droplets are mislabeled, in the sense that they are in fact doublets labeled as singlets. These can mislead the classifier. For this reason, classification is performed in an iterative fashion: at each round, the droplets confidently identified as doublets are removed from the training data for the next round. Similarly, when using randomly-generated (as opposed to between-cluster) artificial doublets, those deemed unidentifiable are removed for the training. We found that 2-3 iterations provided the best performance ( *Extended data* – Figure 1B).

A previous version of *scDblFinder* was already evaluated in an independent benchmark by Xi and Li (2021a), where it was found superior to existing alternatives across a variety of metrics. Here we reproduced this benchmark using the most recent versions of the packages, and including variant methods from the *scDblFinder* package (among which the updated version of scran’s original method, and now available in the *scDblFinder* package as *computeDoubletDensity*). In addition, we included the new method Chord ( Xiong *et al.* 2021), which also combines different strategies.

**Figure 3.**
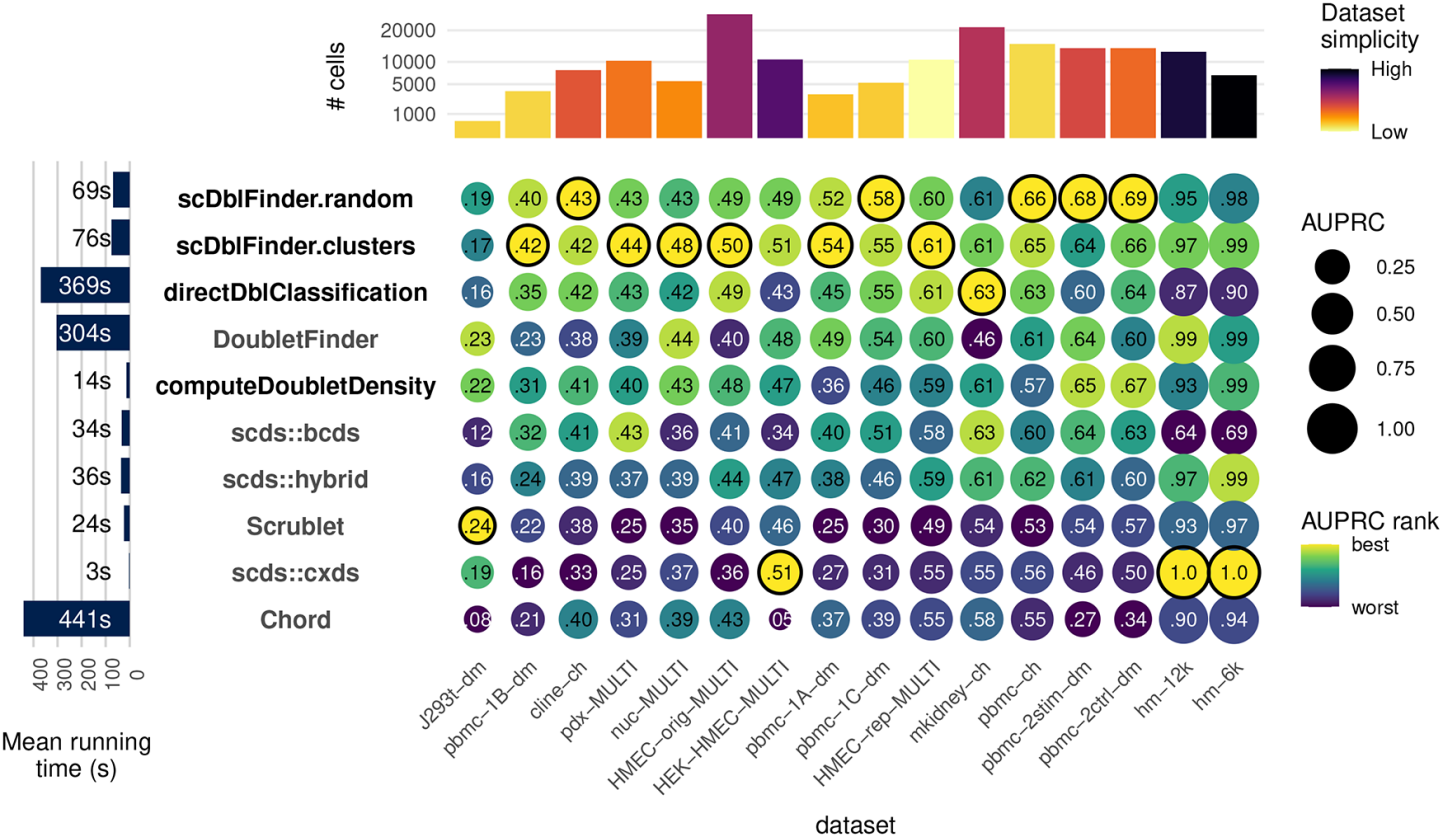
Benchmark. Accuracy (area under the precision and recall curve) of doublet identification using alternative methods across 16 benchmark datasets. The colour of the dots indicates the relative ranking for the dataset, while the size and numbers indicate the actual area under the (PR) curve. For each dataset, the top method is circled in black. Methods with names in black are provided in the *scDblFinder* package. Running times are indicated on the left. On top the number of cells in each dataset is shown, and colored by the proportion of variance explained by the first two components (relative to that explained by the first 100), as a rough guide to dataset simplicity.

Figure 3 compares the performance of *scDblFinder* to alternatives across the real benchmark datasets, as measured by the area under the precision-recall (PR) curve (AUPRC) in classifying annotated doublets. *scDblFinder* is the top performer, except for very simple datasets where most methods perform very well, and one dataset where they all perform badly (and which we suspect not to have enough data for training). Calculating the mean AUPRC across the datasets, the top two methods are the two *scDblFinder* variants ( *Extended data* – Figure 1C). In addition, *scDblFinder* runs at a fraction of the time required by the next best methods ( Figure 3, left).

### kNN summarization improves upon direct classification

*scDblFinder* and the *bcds* method ( Bais and Kostka 2020) are both based on a boosted classifier trained on artificial doublets, however *scDblFinder* performs considerably better. We hypothesized that this improvement would come from two main sources. First, an improvement of *scDblFinder* is the iterative training, which prevents doublets among the real droplets (which are wrongly annotated as singlets) from misleading the classifier. The observed impact of the iterative procedure ( *Extended data* - Figure 1B) however suggests that it explains only part of the difference in performance. Another important difference is that while *bcds* trains directly on the expression matrix, *scDblFinder* works chiefly on features of the kNN network. Since artificial doublet creation can only approximate real doublets, we hypothesized that these differences are more likely to be apparent in the expression matrix than in the highly summarized set of features used by *scDblFinder*, and that this could lead to overfitting on the artificial problem. Indeed, a risk of classifier-based approaches is that the problem on which the classifier is trained, namely distinguishing *artificial* doublets from *real* droplets, slightly differs from the real problem on which they are expected to function (distinguishing *real* doublets from *real* singlets). To investigate this hypothesis of overfitting, we implemented a version of *scDblFinder* without the dimensionality reduction and kNN steps, and training the classifier directly on the expression of the selected genes (see Direct classification). This resulted in a reduction in area under the precision and recall curve (AUPRC) in real datasets (see also Figure 3 and *Extended data* – Figure 2). We therefore conclude that, while dimensionality reduction and kNN summarization arguably involve a loss of information, it nevertheless increases accuracy (in addition to considerably reducing computing time), presumably by preventing overfitting.

### *scDblFinder* identifies most heterotypic doublets

**Figure 4.**
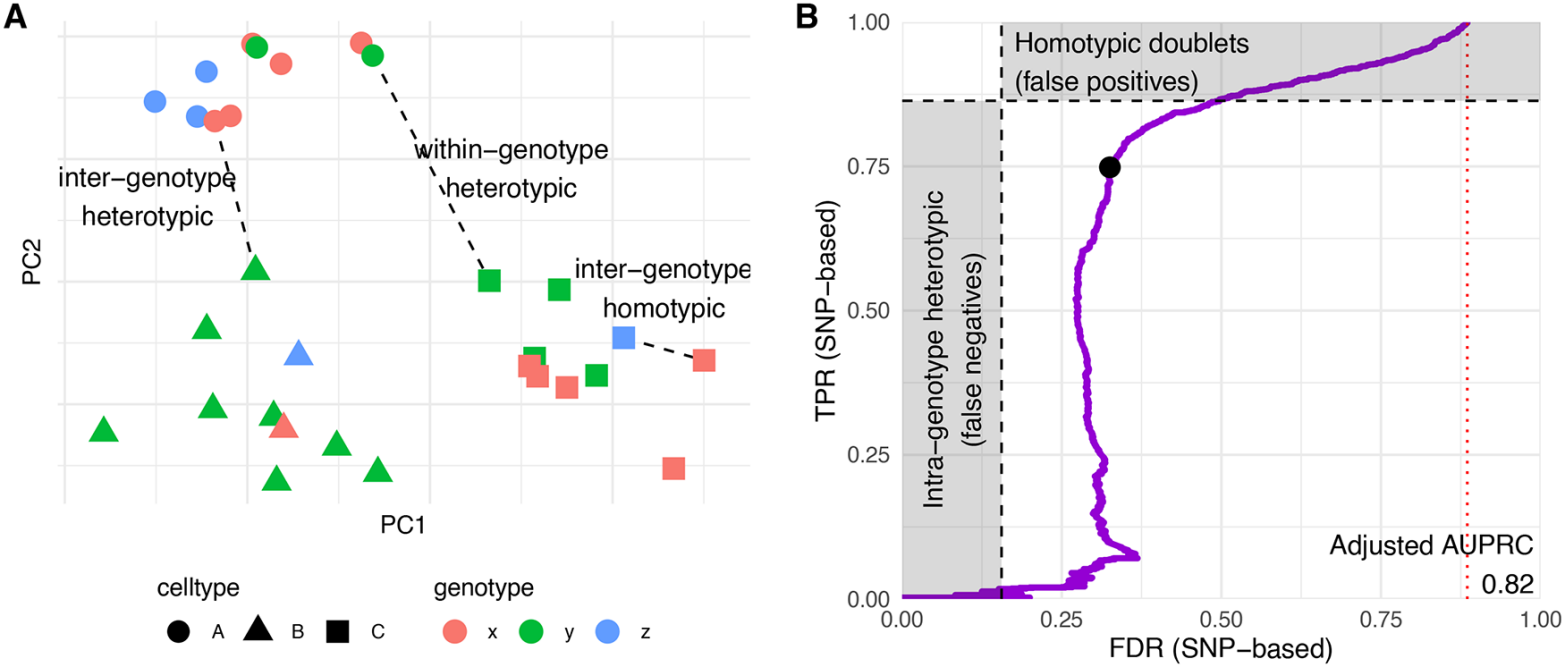
Doublet types and real accuracy of heterotypic doublet identification. A: Cartoon representing the different types of doublets. Within-individual heterotypic doublets will wrongly be labeled as false positives, and between-individual homotypic will be labeled as false negatives. B: Adjusted PR curve for an example sample (GSM2560248). The two shaded areas represent the expected proportion of within-individual heterotypic doublets (i.e. wrongly labeled as singlets in the annotation used as ground truth) and between-individual homotypic doublets, respectively. The red dotted line indicates the random expectation, and the black dot indicates the threshold set by *scDblFinder.*

Several of the benchmark datasets have known doublets flagged by mixing of single-nucleotide polymorphisms from multiple individuals ( Kang *et al.* 2018). In most of these cases, however, such annotation is an imperfect ground truth, for two reasons ( Figure 4A, see also McGinnis, Murrow and Gartner 2019). First, the doublets include also between-individual homotypic doublets, i.e. doublets formed by cells of the same type from different individuals. These are difficult to detect from gene expression, and are arguably of lower priority since they resemble real cells more closely. More importantly, SNPs-based labels do not include heterotypic doublets that are the result of the combination of different cell types from the same individual. It is therefore likely that the accuracy reported in the benchmark is below the actual one in detecting heterotypic doublets, and indeed datasets where there is a full correspondence between cell type and individual (such as the human-mouse mixtures hm-6k and hm-12k) typically have a much higher area under the Receiver-operator characteristic (ROC) and precision-recall (PR) curves ( Figure 3). Based on the frequency of the different individuals and cell types in a dataset, it is possible to infer the expected rate of between-individual homotypic doublets and within-individual heterotypic doublets. This, in turn, enabled us to estimate the performance in identifying *heterotypic* doublets, as opposed to inter-individual but homotypic doublets. Figure 4B shows such an analysis for a complex dataset from Kang *et al.* (2018). The inflection point of the PR curve roughly coincides with the expected proportion of heterotypic doublets among those flagged as true doublets.

Adjusting for both types of ‘misannotation’ (i.e. homotypic doublet and missed within-individual doublets), the area under the PR curve is considerably better (0.82 instead of 0.64), and at the automatic threshold we estimate that 87% of heterotypic doublets can be identified with a real FDR of 17% (a similar analysis for a different sample is shown in *Extended data* – Figure 3).

### Flexible thresholding for doublet calling

Most doublet detection methods provide a ‘doublet score’ that is higher on average in doublets than in singlets, and users are left to decide on a threshold above which droplets will be excluded as doublets. Different methods have been suggested to this end. Building on the fairly tight relationship (especially in 10x-based datasets) between the number of cells captured and the rate of doublets generated ( Kang *et al.* 2018), some have set thresholds based on the number of doublets (or heterotypic doublets) one expects to find in the data ( McGinnis, Murrow, and Gartner 2019). Others have used the best tradeoff in misclassifying artificial doublets from real droplets ( Wolock, Lopez, and Klein 2019). Because *scDblFinder*’s scores come from a classifier, they are analogous to this tradeoff, and can directly be interpreted as a probability (not adjusting, however, for the base rate of doublets).

**Figure 5.**
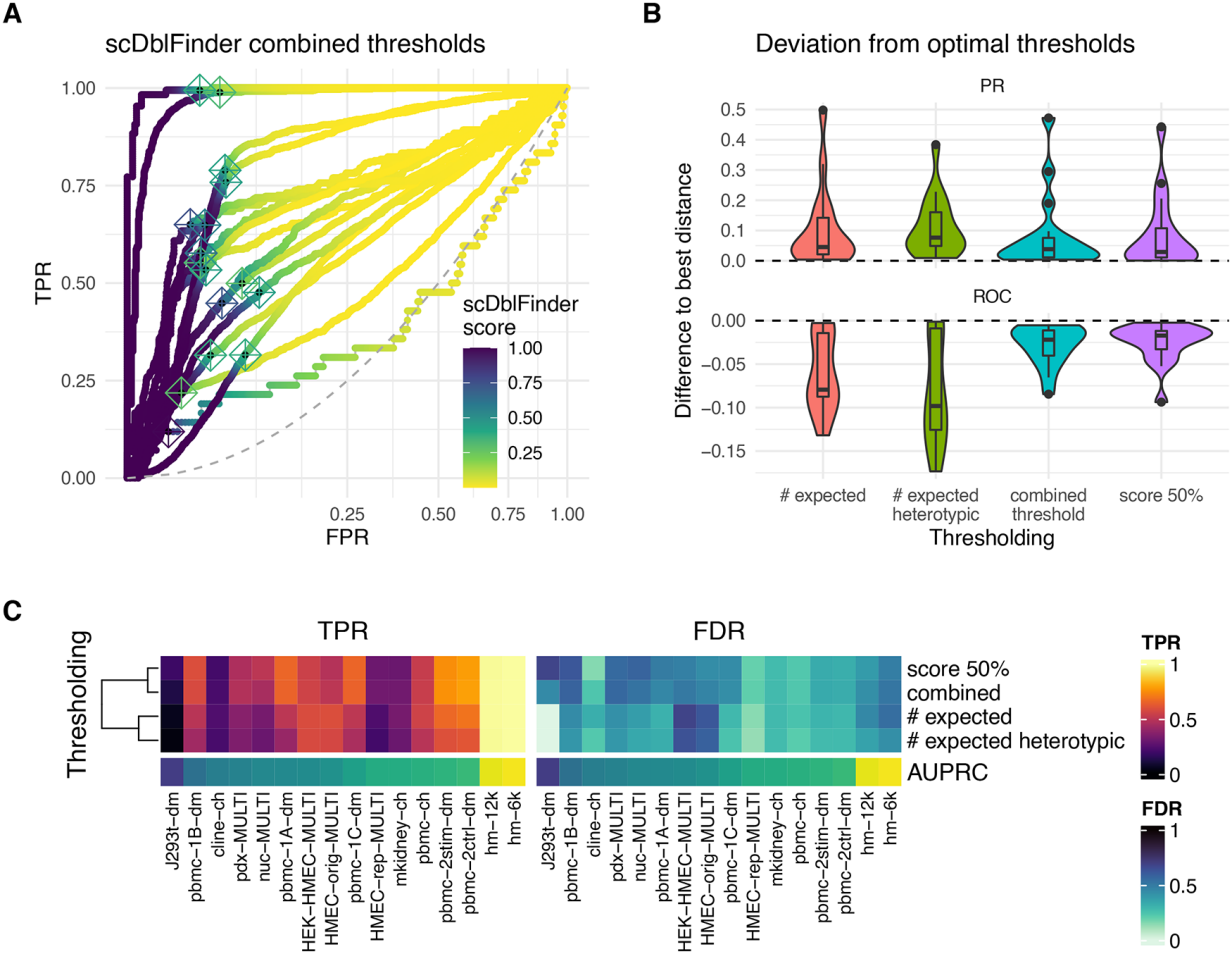
Thresholding. A: ROC curves (with square-root transformation on the x axis) of the different benchmark datasets, colored by *scDblFinder* doublet scores, showing a rapid flip of the scores around the inflexion point. The crosses indicate the *scDblFinder* thresholds. B: Deviation from two ideals of thresholds based on different methods. In the PR curve, the ideal is defined as the minimal distance from the corner indicating a perfect precision and recall. In the ROC curve, the ideal is defined as the maximal distance from the diagonal. The y-axis indicates the difference between the distance at the threshold and the respective optimal distance. C: Tradeoff between True Positive Rate (TPR/sensitivity/recall) and False Discovery Rate (FDR/1-precision) using different thresholds.

With true labels available, the benchmark datasets can again be used to evaluate thresholds. In most cases, we found the *scDblFinder* scores to change rapidly from high to low very close to the inflection point of the ROC curve ( Figure 5A), indicating that a fixed threshold (e.g. 0.5) can often be used. In some cases, the scores are much more gradual, requiring a non-arbitrary way to set the thresholds. *scDblFinder* therefore includes a thresholding method that combines both of the aforementioned rationales, and attempts to minimize both the proportion of artificial doublets being misclassified and the deviation from the expected doublet rate (see Thresholding and *Extended data* – Figure 4A).

Ideal thresholds defined by the ROC and PR curves, while not normally available in practice, can be used here to compare the different thresholding procedures. The optimum represented by the elbow of the ROC curve gives equal weight to the *rate* of both types of errors; however, due to the lower frequency of doublets, in absolute terms this amounts to considering a missed doublet worse that a wrongly excluded singlet. Another ideal threshold can be defined from the PR curve, as the shortest distance to the corner defined by a perfect precision and recall. This second optimum gives a more balanced weight to cells misclassified in one fashion or the other. Figure 5B compares the different thresholding procedure with respect to their deviation from both of these ideals. The fixed score threshold and the *scDblFinder* combined threshold provide similar results, and are both clearly superior (with respect to both ideals) to thresholds based solely on the expected doublet rate. Figure 5C shows the TPR and FDR at each of the computed thresholds across datasets.

### Doublet detection across multiple samples/captures

**Figure 6.**
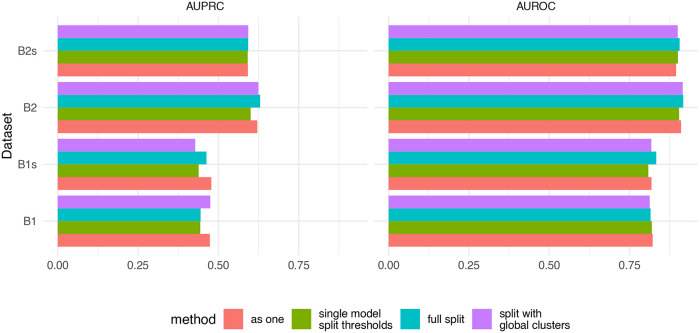
Comparison of four multi-sample strategies. B1 and B2 the two batches from dataset GSE96583, and contain 3 and 2 captures, respectively. The datasets with the suffix ‘s’ are versions downsampled to 30%. Using doublet detection on each capture separately (full split) was generally comparable to treating the captures as one (and adjusting the doublet rate).

Multiple samples are often profiled and analyzed together, with the very common risk of batch effects (either technical or biological) across samples ( Lütge *et al.* 2021). Therefore, while the droplets from all samples might in principle provide more information for doublet detection than a single sample can afford on its own, this must be weighted against the risk of bias due to technical differences. To investigate this, we implemented different multi-sample approaches and tested them on two real multi-sample datasets with demuxlet-based true doublets, as well as a sub-sampling of them ( Figure 6).

The different multi-sample strategies had only a minor impact on the accuracy of the identification. Based on these results, one could take the best overall strategy to be to process all samples as if they were one, however in our experience this can lead to biases against some samples when there are very large variations (e.g. in number of cells or coverage) across samples (not shown). This approach also greatly increases running time. In contrast, running the samples fully separately is computationally highly efficient, and is often equally accurate. This being said, more multi-sample datasets with ground truth will be needed to establish the optimal procedure.

### Feature aggregation enables the use of *scDblFinder* on scATACseq

**Figure 7.**
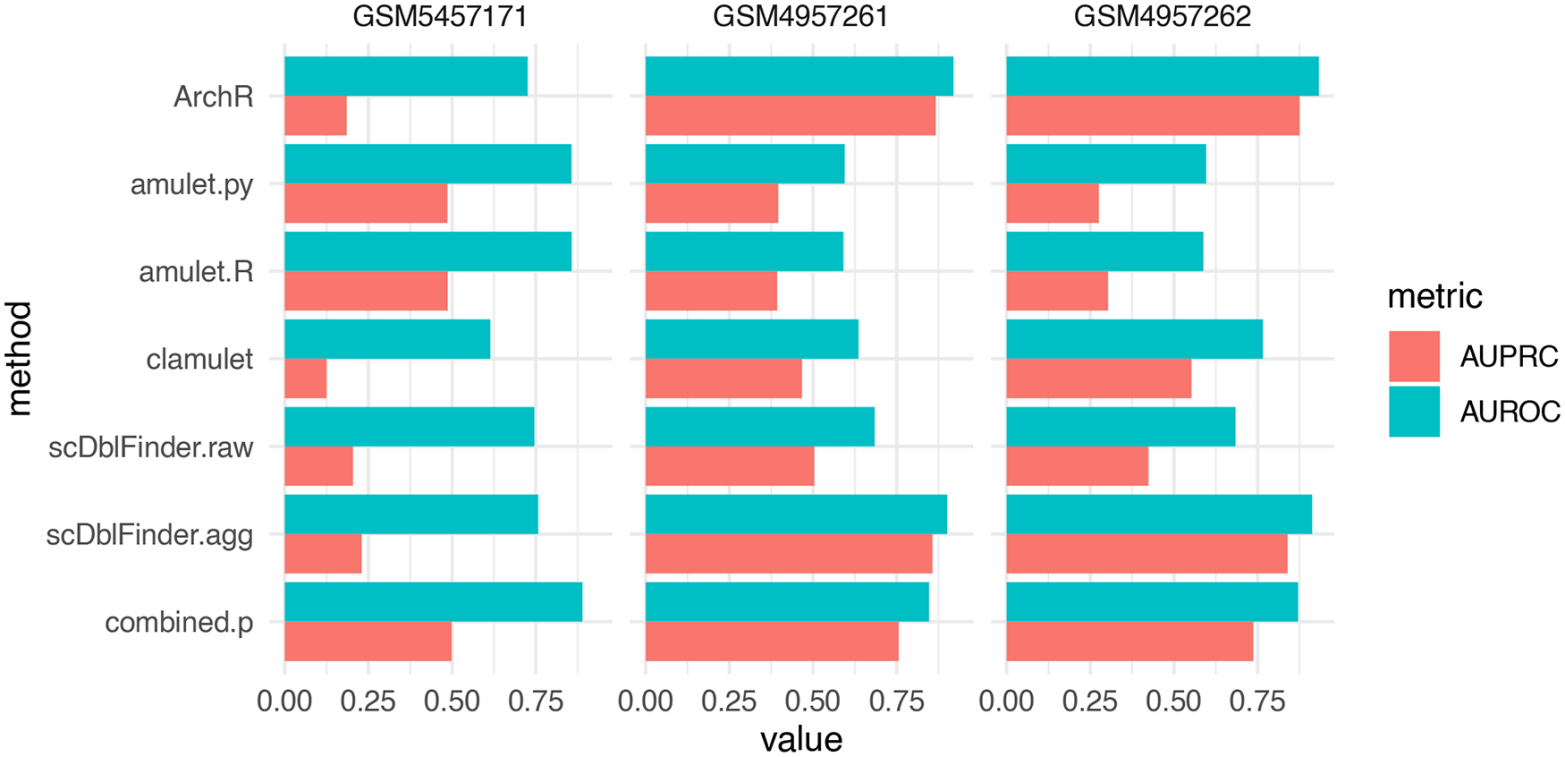
Doublet identification in three single-nucleus ATAC-seq datasets. ‘amulet.py’ and ‘amulet. R’ respectively stand for the original and R reimplementation of the method. ‘scDblFinder.agg’ stands for the feature aggregation approach. ‘combination’ indicates a Fisher combination of the amulet. R p-value and the 1 minus the scDblFinder.agg score. For ‘ArchR,’ the DoubletEnrichment output was used.

We next investigated whether *scDblFinder* could be applied to other types of single-cell data prone to doublets, such as single-cell Assay for Transposase-Accessible Chromatin sequencing (ATACseq). We compared *scDblFinder* to two methods specifically designed to scATACseq: the *ArchR* package ( Granja *et al.* 2021), which implements a doublet detection method that is also based on the comparison to artificial doublets, and the *AMULET* method ( Thibodeau *et al.* 2021). *AMULET* is based on the assumption that, in a diploid cell, any given genomic region should be captured at most twice, and therefore interprets a larger number of loci with more than two reads as indicative of the droplet being a doublet. Since it was only available in the form of a mixture of java and python scripts, we re-implemented the method in the *scDblFinder* package, leading to highly comparable results ( Figure 7). Of note, the *AMULET* method has the advantage of capturing homotypic doublets, which tend to be missed by other methods.

The methods were compared across three datasets where a genotype-based annotation was available as ground truth: two obtained from Granja *et al.* (2021; GSM4957261 and GSM4957262), which by design do not have homotypic doublets, and the dataset published along *AMULET* (GSM5457171; Thibodeau *et al.* 2021). The latter contains homotypic doublets, but its doublet annotation is highly incomplete: due to the low number of individuals multiplexed, we expected to have approximately 35% of the doublets within-individual, and hence mislabeled as singlets.

With default parameters, *scDblFinder* performed very poorly ( Figure 7). This is chiefly because *scDblFinder* follows the common scRNAseq strategy of selecting an informative subset of the features, while ATACseq reads are typically sparsely distributed across the genome. However, working with all features (i.e. peaks) is computationally very expensive. An alternative to both approaches is to begin by reducing the size of the dataset by *aggregating* correlated features into a relatively small set, thereby using information from all. These aggregated features can then directly be used as the space in which to calculate distances. This method yielded comparable performance to specialized single-cell ATACseq software ( Figure 7).

While of an elegant simplicity, the *AMULET* approach performed well only on one dataset ( Figure 7). However, the authors indicate that larger library sizes are needed for the approach to perform well, which is not the case for most droplets in these datasets. Another problem is that the number of loci with more than two reads is strongly dependent on library size ( *Extended data* - Figure 5), however this dependency cannot easily be taken into account because ATAC doublets also tend to have a larger library size, making the two variables confounded.

Since none of the methods appeared clearly superior across all datasets, we next investigated two ways of combining the logics of *scDblFinder* (aggregation) and *AMULET.* First, we developed the *clamulet* method (for classifier-powered Amulet), which mimics the *scDblFinder* workflow but creates artificial doublets from coverages, enabling the use, as part of the predictions, the number of loci covered by more than two reads. We also tried running both methods separately and aggregating the resulting *p*-values using Fisher’s method (which proved better than averages or rank-based aggregation). This proved to be the most satisfactory approach, providing result that are more robust across datasets ( Figure 7). This being said, more complex ways of aggregating calls from different methods could be explored ( Xiong *et al.* 2021; Neavin *et al.* 2022), and more work, and especially on a broader set of benchmark datasets, will be necessary to establish optimal methods.

### Doublet origins and enrichment analysis

When artificial doublets are generated between clusters, we know which clusters constitute them, and we reasoned that this information could be used to infer the clusters composing real doublets (hereafter referred to as ‘doublet origin’). Using a simulation as well as the aforementioned real dataset with doublets of known origins (mixture of five cell lines from Tian *et al.* (2018)), we first assessed the accuracy of doublet origin prediction based on the nearest artificial doublets in the kNN. These proved inaccurate, both in real and simulated data (see *Extended data* – Figure 6A-B). Even training a classifier directly on this problem failed (see *Extended data* – Figure 6C-D). The problem appears to be that, due to the very large variations in library sizes (and related variations in relative contributions of the composing cells – see Figure 1B), doublets often contain a large fraction of reads from one cell type, and conversely a small fraction from the other cell type. As a consequence, we can typically call at least one of the two originating cell types, but seldom both. In the real dataset, at least one of the two originating cell types is correctly identified in 73% of doublets (random expectation: 36%), but both are correct in only 20% of cases.

**Figure 8.**
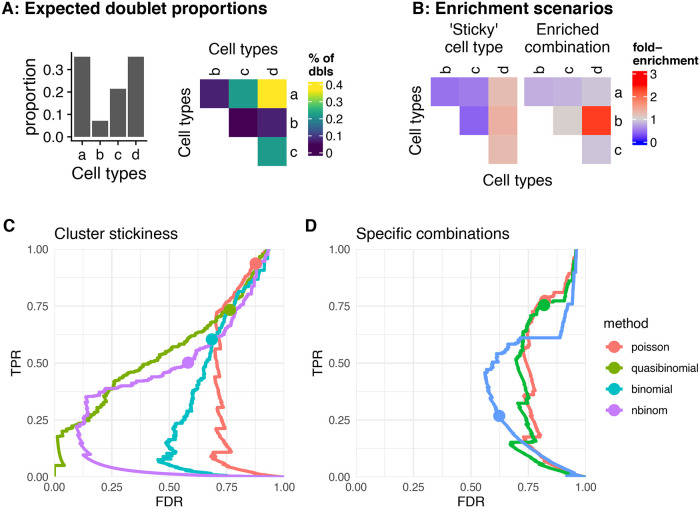
Doublet enrichment analysis. A, B: Doublet enrichment in a toy example. A: Proportion of different doublet types from random expectations based on the cell type abundances. B: The fold-enrichment over this expectation in two different doublet enrichment scenarios. C, D: Performance of the cluster stickiness tests (C) and tests for enrichment of specific combinations (D) using different underlying distributions.

While the identification of doublet origins remains a challenge, for the sake of completeness we nevertheless developed strategies to investigate whether certain doublet types were found more often than expected. Such enrichment could, for instance, indicate cell-to-cell interactions. We defined two forms of doublet enrichment ( Figure 8A- B), and specified models to test each possibility: i) enrichment in doublets formed by a specific combination of celltypes, or ii) enrichment in doublets involving a given cell type, denoted ‘sticky.’

The ‘stickiness’ of each cluster (as proxy for cell types) can be evaluated by fitting a single generalized linear model on the observed abundance of doublets of each origin (see Methods). We tested the performance of this test under different underlying distributions using simulated doublet counts. The number of doublets of each type is generated from random expectation with or without added stickiness (as factors of 1 to 3 on the probability) using negative binomial distributions with different over-dispersion parameters ( Figure 8C and *Extended data* – Figure 7). The quasi-binomial showed the best performance, followed by the negative binomial, but in all cases the p-values were not well calibrated and many false positives were reported at a nominal FDR<0.05. This was robust across different over-dispersion values (see *Extended data* – Figure 7).

We next sought to establish a test for the enrichment of specific combinations. Here, we simply computed the probability of the observed counts for each combination using different models (see Methods). We again tested this approach relying on different underlying distributions, on simulations with varying over-dispersion. The negative binomial performed best, however all variants suffered a high false discovery rate ( Figure 8C).

## Conclusions

The *scDblFinder* package includes a set of efficient methods for doublet detection in both single-cell RNA and ATAC sequencing. In particular, the main *scDblFinder* approach uses integrates insights from previous approaches into a comprehensive doublet detection method that provides robustly accurate detection across a number of benchmark datasets, at a considerably greater speed and scalability than the best alternatives. Even in complex datasets, most heterotypic doublets can be accurately identified. Although the doublet scores given by *scDblFinder* can be directly interpreted as probabilities, simplifying their interpretation, the method also includes a trade-off thresholding procedure incorporating doublet rate expectations with classification optimization, thereby facilitating its usage.

*scDblFinder* additionally provides utilities for identifying the origins of doublets (in terms of composing cell types) and testing for different forms of doublet enrichment. At present, however, the value of such tests is limited by the difficulty of accurately identifying doublet origins. Further research will be needed to assess to what extent this can be improved.

In conclusion, we believe that *scDblFinder*, with its flexibility, accuracy and scalability, represents a key resource for doublet detection in high-throughput single-cell sequencing data.

## Methods

### scDblFinder implementation

As a first step, the dataset is reduced to its top most expressed features (1000 by default); if the cluster-based approach is used, the top features per cluster are instead selected.

The generation of artificial doublets then depends on whether the clustered or random mode is used. If using the cluster-based approach (and not manually specifying the clusters), a fast clustering is performed (see Fast clustering). Artificial doublets are then created by combining random droplets of different clusters, proportionally to the cluster sizes. In explicitly concentrating on between-cluster doublets, we do not attempt to identify homotypic doublets, which are anyway virtually unidentifiable and relatively innocuous. In doing so, we reduce the necessary number of artificial doublets (since no artificial doublet is ‘lost’ modeling homotypic doublets), and prevent the classifier from being trained to recognize doublets that are indistinguishable from singlets, which would lead to calling singlets as doublets.

An alternative strategy also available in *scDblFinder* is to generate fully random artificial doublets, and use the iterative procedure (see below) to exclude unidentifiable artificial doublets from the training. In practice, the two approaches have comparable performances ( Figure 3), and they can also be combined.

Dimension reduction is then performed on the union of real and artificial droplets, and a nearest neighbor network is generated. The network is then used to estimate a number of characteristics for each cell, in particular the proportion of artificial doublets among the nearest neighbors. Rather than selecting a specific neighborhood size, the ratio is calculated at different values of *k*, creating multiple predictors that will be used by the classifier. A distance-weighted ratio is also included. Further cell-level predictors are added, including: projections on principal components; library size; number of detected features; and co-expression scores (based on a variation of Bais and Kostka 2020). *scDblFinder* then trains gradient boosted trees to distinguish, based on these features, artificial doublets from real droplets. Finally, a thresholding procedure decides the score at which to call a droplet by simultaneously minimizing the misclassification rate and the expected doublet rate (see Thresholding).

*Artificial doublet generation*

For artificial doublet generation, only droplets with library sizes with the 5-95 percentiles are used. Doublets are then created using random pairs of droplets (or random between-cluster pairs, if clusters are used). The majority (75% by default) are generated by summing the counts of the two droplets. For the remaining the sum is divided by two and used as mean for Poisson sampling to yield counts. If clusters are used, for half of these last doublets the contributions of the two droplets are also re-weighted using the clusters’ median library size: rather than the two droplets contributing to the doublet based on the ratio of their actual library size, this is averaged with the ratio of the median library size of their respective cluster.

*Parameter optimization*

Using the benchmark datasets from Xi and Li (2021a), we next optimized a number of parameters in the procedure, notably regarding features to include and hyperparameters, so as to provide robust default parameters (see *Extended data* – Figures 8-11). Some features, such as the distance to the nearest doublet or whether the nearest neighbor is an artificial doublet, had a negative impact on performance (see *Extended data* – Figure 8), presumably because it led to over-fitting. Finally, in line with a discrepancy between the trained and real problems, we observed that the variable importance calculated during training (see *Extended data* – Figure 9) did not necessarily match that of the variable drop experiments (see *Extended data* – Figure 8).

We finally optimized learning hyperparameters (see *Extended data* – Figure 10) and further input parameters (see *Extended data* – Figure 11).

*Fast clustering*

Irlba-based singular value decomposition is first run using the *BiocSingular* package, and a kNN network is generated using the Annoy approximation implemented in *BiocNeighbors* . Louvain clustering is then used on the graph. If the dataset is sufficiently large (>1000 cells), a first rapid k-means clustering (using the mbkmeans package) is used to generate a large number of meta-cells, which are then clustered using the graph-based approach, propagating clusters back to the cells themselves.

*Thresholding*

Unless manually given, the expected number of doublets ( *e*) is specified by *e* = *n* ^2^/10 ^−5^ (where *n* is the number of cells captured). This is then restricted to heterotypic doublets using random expectation from cluster sizes or, if not using the cluster-based approach, using the proportion of artificial doublets misidentified. The doublet rate is accompanied by an uncertainty interval ( *dbr.sd* parameter), and the deviation from the expected doublet number for threshold *t* is then calculated as

$$\text{deviatio}n_{t}=\left\{\begin{array}{ll} 0 & \text{if}\left(o_{t},\geq ,e_{\mathrm{low}},\wedge ,o_{t},\leq ,e_{\mathrm{high}}\right)\\ 2\cdot \frac{\text{min}\left(\left|o_{t},-,e_{\mathrm{low}}\right|,,,\left|o_{t},-,e_{\mathrm{high}}\right|\right)}{e_{\mathrm{low}}+e_{\mathrm{high}}} & \text{otherwise} \end{array}\right.$$

where *o _t_* represents the number of real cells classified as doublets at threshold *t*, and *e _low_* and *e _high_* represent, respectively, the lower and higher bounds of the expected number of heterotypic doublets in the dataset (based on the given or estimated doublet rate the *dbr.sd* parameter). The default value of the *dbr.sd* parameter was roughly estimated from the variability of observed doublet rates ( *Extended data* – Figure 4B). The cost function being minimized is then simply given by *cost*_*t*_ = *FNR*_*t*_ + *FPR*_*t*_ + *deviation*_*t*_^2^, where the false negative rate ( *FNR _t_*) represents the proportion of artificial doublets misclassified as singlets at threshold *t*, and the false positive rate ( *FPR _t_*) represents the proportion of real droplets classified as doublets. This is illustrated in *Extended data* (Figure 4A).

Since this is performed in an iterative fashion, the FPR is calculated ignoring droplets which were called as doublets in the previous round.

### Doublet enrichment analysis

*Cluster stickiness*

Cluster ‘stickiness’ can be evaluated by fitting a single generalized linear model on the observed abundance of doublets of each origin, in the following way:

$$\log\left({\text{observed}}_{i}+0.1\right)=\log\left(e_{i}\right)+\beta_{z}\cdot \log\left({\text{difficulty}}_{i}\right)+\beta_{a}a_{i}+\beta_{b}b_{i}+\beta_{c}c_{i}+\ldots +\epsilon_{i},$$

where *observed*_*i*_ and *e*_*i*_ represent the numbers of doublets formed by specific combination *i* of clusters which are respectively observed or expected from random combinations, and *a _i_*, *b _i_* and *c _i_* (etc) indicate whether or not (0/1) the doublet involves each cluster.

Because some doublets are easier to identify than others, some deviation from their expected abundance is typically observed. For this reason, a *difficulty _i_* term is optionally included, indicating the difficulty in identifying doublets of origin *i*, which is the rate of misclassification of *scDblFinder*’s artificial doublets of that origin (by default, the term is included if at least 7 clusters are present). A *β*_*a*_ significantly different from zero, then, indicates that cluster *a* forms more or less doublets than expected – if positive, it indicates cluster ‘stickiness.’

For the (quasi-)binomial distributions, logit was used instead of log transformation, and the mean of observed and expected counts was used as observational weights.

*Enrichment for specific combinations*

To account for the different identification difficulty across doublet types, we first fit the following global negative binomial model:

$$\log\left(\text{observe}d_{i}\right)=\alpha +\log\left(e_{i}\right)+\beta \cdot \log\left(\text{difficult}y_{i}\right),$$

where *observed _i_* and *expected _i_* are respectively the observed and theoretically expected number of doublets of type *i*, and the *difficulty _i_* term is the same as for the stickiness problem above. Then, the fitted values are then considered the expected abundance, and a p-value for each doublet type is given by the probability of the observed count under this adjusted expected value, using either distribution (for the negative binomial, the global over-dispersion parameter calculated in the first step is used).

### Direct classification

The direct classification approach is implemented in the *directDblClassification* function of the package. It uses the same doublet generation, thresholding and iterative learning procedures as *scDblFinder*, but trains directly on the normalized expression matrix of real and artificial cells instead of kNN-based features. The hyperparameters were the same except for the maximum tree depth, which was increased to six to account for the increased complexity of the predictors.

### Feature aggregation

For feature aggregation (used for scATACseq), *scDblFinder* first normalizes the counts using the Term Frequency - Inverse Document Frequency (TF-IDF) normalization, as implemented in Stuart *et al.* (2019). PCA is then performed and the features are clustered into the desired number of meta-features using mini-batch k-means ( Hicks *et al.* 2021) or, if not available, simple k-means. The counts are then summed per meta-feature.

### Benchmark

*Datasets*

We used the scRNAseq benchmark datasets prepared by Xi and Li (2021a), which were originally published by Kang *et al.* (2018), Stoeckius *et al.* (2018), McGinnis, Murrow, and Gartner (2019), McGinnis *et al.* (2019), and Wolock, Lopez, and Klein (2019).

*Metrics*

The area under the PR or ROC curves were calculated using integral method, implemented in the PRROC package ( Grau, Grosse, and Keilwagen, 2015). The adjusted AUPRC, meant to capture the AUPRC accounting for homotypic and within-individual doublets, was calculated as the proportion of the unshaded area in Figure 4. Specifically, values were linearly scaled values so that an observed FDR of corresponding to the expected proportion of within-individual doublets is set to 0, and that an observed TPR corresponding to one minus the expected proportion of homotypic doublets as an adjusted TPR of 1. Values were capped to be within a 0-1 range, and the area under the curve was calculated using trapezoid approximation. The expected proportion of homotypic doublets was estimated using the clusters from the fast clustering method described above (see Fast clustering).

The reported metrics are an average of the results of two runs using different random seeds.

### scDblFinder operation

*scDblFinder* is provided as a bioconductor package. The input data for *scDblFinder* (denoted *x* below) can be either i) a count matrix (full or sparse), with genes/features as rows and cells/droplets as columns; or ii) an object of class *SingleCellExperiment* . In either case, the object should not contain empty drops, but should not otherwise have undergone very stringent filtering (which would bias the estimate of the doublet rate). The doublet detection can then be launched with:

*library (scDblFinder)*

*sce <- scDblFinder(x)*

The output is a SingleCellExperiment object including all of the input data, as well as a number of columns to the *colData* slot, the most important of which are:

• *sce$scDblFinder.score*: the final doublet score (the higher the more likely that the cell is a doublet)
• *sce$scDblFinder.class*: the binary classification (doublet or singlet)

scDblFinder can run on any system running R >= 4.0 and Bioconductor >= 3.12.

For more details, see the package’s vignettes.

## Software availability

*scDblFinder* is available from Bioconductor: http://www.bioconductor.org/packages/release/bioc/html/scDblFinder.html.

The source code is available from: https://github.com/plger/scDblFinder.

Archived source code at time of publication: https://doi.org/10.6084/m9.figshare.16543518 ( Germain 2021a).

The software is released under the GNU Public License (GPL-3).

## Data availability

### Underlying data

figshare: scDblFinder. https://doi.org/10.6084/m9.figshare.16543518 ( Germain 2021a).

This repository contains the following underlying data:

• scDblFinder 1.9.12 (archived software version used in the paper).
• scDblFinder_paper (code to reproduce the analyses and figures).

The code to reproduce the analyses and figures is additionally available at https://github.com/plger/scDblFinder_paper.

Data are available under the terms of the Creative Commons Attribution 4.0 International license (CC-BY 4.0).

### Extended data

figshare: Supplementary Figures for the scDblFinder paper.

https://doi.org/10.6084/m9.figshare.16617571 ( Germain, 2021b)

This repository contains the following extended data:

• Supplementary Figures 1-10

Data are available under the terms of the Creative Commons Attribution 4.0 International license (CC-BY 4.0).
