# Supplementary material for: Doublet identification in single-cell sequencing data using scDblFinder
Source: F1000Res. 2022 May 16;10:979. Originally published 2021 Sep 28. [Version 2] doi: 10.12688/f1000research.73600.2 (PMC9204188; doi:10.12688/f1000research.73600.2)
Supplement: Peer review history

# Peer review: Reviewer response for version 2

**DOI:** 10.5256/f1000research.133464.r138015

**Published:** 2022-06-16

**Authors:** Zev J. Gartner, Chris McGinnis

**Affiliations:** 1 Department of Pharmaceutical Chemistry, University of California, San Francisco, San Francisco, CA, USA; 2 University of California, San Francisco, CA, USA

The authors have addressed all of our concerns and have really improved the readability of the manuscript. This is fantastic work.

Are the conclusions about the tool and its performance adequately supported by the findings presented in the article?

Yes

Is the rationale for developing the new software tool clearly explained?

Yes

Is the description of the software tool technically sound?

Yes

Are sufficient details of the code, methods and analysis (if applicable) provided to allow replication of the software development and its use by others?

Yes

Is sufficient information provided to allow interpretation of the expected output datasets and any results generated using the tool?

Yes

Reviewer Expertise:

Tissue engineering and single cell analysis method development

We confirm that we have read this submission and believe that we have an appropriate level of expertise to confirm that it is of an acceptable scientific standard.

# Peer review: Reviewer response for version 1

**DOI:** 10.5256/f1000research.77262.r95560

**Published:** 2021-11-08

**Authors:** Dennis Kostka, Hannah Schriever

**Affiliations:** 1 Department of Developmental Biology; Department of Computational & Systems Biology; Pittsburgh Center for Evolutionary Biology and Medicine, University of Pittsburgh School of Medicine, Pittsburgh, PA, USA; 2 Department of Developmental Biology, University of Pittsburgh School of Medicine, Pittsburgh, PA, USA; 3 Joint CMU-Pitt Ph.D. Program in Computational Biology, Pittsburgh, PA, USA

**Summary**

 The authors describe scDblFinder, a computational tool for doublet/multiplet annotation in single-cell RNA sequencing and single-cell ATAC sequencing data. They explain and motivate method design and parameter choices, reproduce a benchmark on scRNA-seq data (originally performed by Xi and Li  2021) and demonstrate scDblFinder’s performance; this includes inferring cell-types of origin for doublets, deriving binarized doublet calls, and annotating doublets scATAC-seq data.

**General Comments**

 The reviewers note that they have not considered other reviews for the same manuscript prior to or during the writing of this report.

 scDblFinder is probably the most competitive method for computational doublet annotation, so this manuscript, describing the method in detail, is an important contribution and valuable for the community.  We have the following general comments:

- Positive points of the manuscript that stand out are the authors’ discussion of their results on annotating cell-types of origin for doublets (and the conclusion that it remains a challenge), scDblFinder’s good performance on scATAC-seq data, and its thresholding approach for generating binarized doublet calls.
- While the quality of the manuscript is generally high, we still feel that clarity can be improved, especially in describing methods (see specific comments).
- Further on, as the authors describe, parameters of scDblFinder have been tuned to maximize performance on 16 benchmark data sets used for evaluation. As a consequence, performance comparison with methods that did not perform parameter optimization on the benchmark data is biased in favor of scDblFinder; this should be mentioned in the manuscript’s discussion section.

**Specific Comments**

- Terminology: It would be good to consistently use a term (like “droplet”, for example) for sequenced units of RNA that can contain more than one cell, whereas the term “cell” could be reserved to indicate a single cell.
- In the introduction, the authors comment on drawbacks of multiplexing technologies and that they only identify a fraction of doublets. It would be good to (a) include that these experimental methods incur additional drawbacks, like increased cost and decreased yield and (b) that there are definite benefits of sample multiplexing in the context of experimental design.
- Methods, page 3: It would be helpful to streamline the description of  scDblFinder's approach/procedure. Specifically, a verbal description would help. In the current version, for example, the authors talk about "the cluster-based approach", but it is not at all clear what they mean at that point in the manuscript.
- Methods, page 4: The authors state that mislabeled cells can mislead the classifier and use this to motivate their iterative approach (in paragraph 2). It would be helpful to reference Supplemental figure 5 here, which shows the positive effect of removing doublet-classified but singlet-annotated droplets.
- Formula on page 5: It is not discussed how the *dbr.sd* parameter that is used for *e_low* and *e_high *is estimated for a given dataset. Is there a motivation for choosing this specific functional form for *deviation*?
- Figure 2: It looks like the dashed line is not at the minimum of the cost function (black line).
- Formulae on page 6 and 8: It is not described how *difficulty* is estimated from misclassification of simulated doublets. In general, it would be good to present a consistent discussion for both models, including motivation, definition of all quantities, and error terms.
- For the dataset used in Figure 3, it would be helpful to give a more in detailed description of the cell lines (organism, type, etc).
- Methods page 9. In paragraph 1, how is the desired number of features determined (is it a method parameter)?
- Results page 10. It would be helpful to briefly describe cell lines in the Tian *et al.* 2018 dataset (see previous comment about Figure 3).
- Results page 11. It would be helpful to add details about how artificial doublets were generated in paragraph 2. How were contributions "re-weighted based on the relative median sizes of the composing cell-types”?
- Results page 11. In paragraph 4 and Figure 5 panel B: How was the expected rate of homotypic / heterotypic doublets estimated? In the legend of Figure 5, maybe change “truth” to “annotation” (also in the short paragraph 5 on page 11). Further on, a description on how the adjusted AUPRC is calculated should be included. Since doublet prediction is imbalanced, has a non-zero minimum AUPRC (https://dl.acm.org/doi/10.5555/3042573.3042780 ^1^) been taken into account as well? Finally, does the black dot in Figure 5 B denote a cutoff such that the number of doublets scDblFinder would call corresponds to the expected number of heterotypic doublets?
- Results page 11. In paragraph 5 (3 lines), it would be helpful to make explicit what two types errors are accounted for. Further on, it appears the adjustment is based on estimated doublet rates, and it would be good to discuss how reliable these are, and how that, in turn, impacts the adjusted areas under the curve. Finally, the reference should be to supplemental figure 8 (not 9).
- Figure 6: It is not clear what the dots in panel A denote. In addition, it is really hard to compare the different points across panels B and C; perhaps sub-panels (one per curve) with both points on each curve would be clearer.
- Results page 11. For the last paragraph, it would be good to provide metrics that compare the two approaches in a qualitative way. One possibility would be a table showing for each benchmark dataset the number of annotated doublets, called doublets, false positives and false negatives together with a performance metric (e.g., Matthews correlation coefficient).
- Results, page 12. The authors conclude from Figure 7 that “as one” is the “best overall strategy”. Performance seems really close (and the conclusion only holds for AUPRC, not really for AUROC), so it is not clear if (a) the advantage is significant and (b) is expected to generalize to other data sets. This could be discussed.
- Figure 8 is very large.
- Figure 9: Some colors are different between panels C and D, which makes interpretation of the legend confusing.

- Supplemental Figure 1: It would be helpful to better explain the setup in the legend, so it is clear what TRUE/FALSE mean and how differences in AUPRC are defined (e.g., high = better performance without variable, low = worse performance without variable). If the violins are summarizing 16 points, it might be informative to show the points themselves. Does the dot indicate the median or the mean, what interval is denoted?
- Supplemental Figure 2: In panel B, maybe show log of elapsed time.
- Supplemental Figure 3: Consider using the same type of violin plot as before.

- Typo on page 5: “based on the given or estimated doublet rate the dbr.sd parameter” should be “based on the given or estimated doublet rate **and** the dbr.sd parameter”.
- Typo on page 8: “probability of each double type” should be “ probability of each double **t** type
- Type on page 11: “This, in turn **s**, allows” should be “This, in turn, allows”

Are the conclusions about the tool and its performance adequately supported by the findings presented in the article?

Yes

Is the rationale for developing the new software tool clearly explained?

Yes

Is the description of the software tool technically sound?

Yes

Are sufficient details of the code, methods and analysis (if applicable) provided to allow replication of the software development and its use by others?

Yes

Is sufficient information provided to allow interpretation of the expected output datasets and any results generated using the tool?

Yes

Reviewer Expertise:

computational genomics, evolutionary genomics, statistical phylogenetics

We confirm that we have read this submission and believe that we have an appropriate level of expertise to confirm that it is of an acceptable scientific standard.

## References

[1] : Unachievable region in precision-recall space and its effect on empirical evaluation. *Omnipress* .2012;1619-1626 Reference source PMC385895524350304

# Response

**Published:** 2022-04-23

**Authors:** Pierre-Luc Germain

We thank the reviewers for their thorough reading and useful comments, and have tried to address all of them in the revised version. A detailed point-by-point version will be made available along with the new version, but here are the main points of response:

1. **Bias due to parameter optimization based on the benchmark datasets: **The reviewers were absolutely right to raise this important point, we now state it clearly in the discussion, with the note however that a previous version of scDblFinder (developed with only 5 of the datasets) was also found to outperform alternatives in the addendum to Xi and Li’s (2020) original benchmark.
2. **Terminology and clarifications: **We thank the reviewers for their excellent suggestions regarding terminology and clarifications, which we adopted in the revised version (see the full point-by-point response for details). We also tried to provide a simpler 'verbal' overview of the method in the Results section, as suggested, and to clarify the methods section.
3. **AUPRC:** We thanks the reviewer for the suggested paper on AUPRC. While it was quite an interesting read, we do not think that either the random or the minimal AUPRC affects any of the claims being made, as it is certainly well below any of those observed, and our main point in this section is that the performance in estimating heterotypic doublets is underestimated in most of the benchmark datasets. This being said, we now added the random expectation in Figure 4B.
4. Finally we also addressed the minor comments.

# Peer review: Reviewer response for version 1

**DOI:** 10.5256/f1000research.77262.r95679

**Published:** 2021-10-19

**Authors:** Zev J. Gartner, Chris McGinnis

**Affiliations:** 1 Department of Pharmaceutical Chemistry, University of California, San Francisco, San Francisco, CA, USA; 2 University of California, San Francisco, CA, USA

**Summary**

 The authors describe scDblFinder — a doublet detection algorithm that uses gradient boosted trees to classify doublets as cells with elevated proportions of artificial nearest neighbors, library sizes, and co-expression scores. The authors describe parameter selection and hyperparameter optimization workflows before benchmark their method against the Xi & Li datasets. These efforts effectively demonstrate that scDblFinder performs very strongly relative to other doublet detection methods. Moreover, building on the existing doublet detection literature, the authors also make the following improvements/alterations to existing workflows and reaffirmations of previous observations: 

  

- Authors simulate doublets using a ‘mixed strategy’ including summing counts, Poisson resampling of counts, and weighted-averaging of counts based on cell-type-specific library sizes. This approach is distinct from current methods which simulate doublets by summing/averaging counts or averaging PC coordinates — Perhaps an improvement but needs more analysis ( **Major comment #1**).

 

- Authors demonstrate that scDblFinder is predominantly sensitive to heterotypic doublets and that removing homotypic doublets from ground-truth data improves doublet prediction performance metrics — Affirming previous observations in the DoubletFinder and other manuscripts. 

 

- Authors threshold doublet classification probability distributions (i.e., likelihood scores generated from gradient boosting) using a combination of two known approaches: (i) Threshold based on artificial doublet misclassification rate (from Scrublet) or (ii) Threshold based on expected number of doublets inferred from droplet microfluidics Poisson loading statistics (from DoubletFinder) — Perhaps an improvement ( **Major comment #2**).

 

- Authors show that scDblFinder (and other classifiers) should be run on individual scRNA-seq samples in instances when those samples are not multiplexed using Cell Hashing, MULTI-seq, etc. — This is an ‘unwritten’ rule for doublet detection workflows but hadn’t been definitively demonstrated. 

 

- Authors show that scDblFinder performs as well as ArchR for predicting doublets in snATAC-seq data after aggregating features — This is good to know, but authors need to include the current gold-standard for snATAC-seq doublet detection in their analysis, AMULET, such that readers are correctly informed about their analytical options for snATAC-seq doublet detection ( **Major Comment #3**).

 

- Authors present a strategy for inferring doublet origins which could be useful for identifying instances of biological doublets formed due to cell-cell interactions or ‘stickiness’ — Could be useful theoretically, but the approach is not sufficiently developed to be useful (although we commend the authors for including this analysis) 

On balance, we believe that scDblFinder is a great method (likely the new gold standard for the field) and strongly recommend its indexing, assuming that our Major Comments are addressed. 

**Major Comments**

  

1. The authors do a good job at justifying the theoretical utility of the proposed ‘mixing strategy’ for doublet simulation in Figure 3. However, while the authors compare scDblFinder performance before/after dimensionality reduction and kNN and using cluster-limited vs random doublet simulation, they do not do analogous analyses for comparing the ‘mixing strategy’ to more standard methods (e.g., summing, averaging, etc.). As such, it is unclear whether the proposed strategy improves performance.
2. In Figure 6, the authors plot ROC curves annotated by the thresholds defined by their proposed cost-minimization framework (Fig. 6B) or by the expected number of doublets (Fig. 6C). Since the cost-minimization framework also aims to minimize artificial doublet misclassification rate (as implemented by Scrublet), we would also like to see the same ROC plots with thresholds defined by this strategy (i.e., instead of just the expected doublet strategy implemented by DoubletFinder). We would also like the authors to provide summary statistics for the comparisons (see *Minor Comment #1*) Without this, it is unclear whether the cost-minimization framework is actually an improvement over existing approaches.  
3. The application of scDblFinder to snATAC-seq data is not satisfactory as it only involves benchmarking against ArchR, which was recently shown in the AMULET paper (Thibodeau, Eroglu, et al. Genome Biology, 2021) to perform sub-optimally on snATAC-seq data. The authors need to include reference to AMULET in this section and benchmark the performance of AMULET, ArchR, and scDblFinder.
4. We believe that the manuscript in its current form suffers from poor readability – we really had to parse every sentence in the Methods section to understand what the authors were intending to convey. For one tangible example, when discussing the overfitting hypothesis on page 4 (which itself was conveyed quite clearly), the authors could include a clear rationale for why comparing scDblFinder performance before and after dimensionality reduction and kNN addresses the hypothesis. Notably, we believe that this manuscript was very well-executed and that scDblFinder is a great method. We also recognize that the authors write very accessibly in the Results section. However, many in the single-cell genomics community who may want to use scDblFinder are not machine learning experts will likely have a hard time appreciating the quality of this work, especially in its current structure (i.e., Methods section before Results). As such, we strongly recommend that the authors add more ‘plain English’ explanations to the Methods section (or put the Results before the Methods section, if the journal will allow that) to improve readability for a broader audience.

**Minor Comments**

1. Should provide quantitative analysis of distance of scDblFinder vs heterotypic doublet thresholding strategies to the real inflection point (related to Figure 6).
2. Authors should cite papers related to the benchmarking datasets used in Xi & Li. ^1^  
3. Authors should cite Chord from Xiong and colleagues, which also uses boosting to improve doublet detection. ^2^
4. On page 3, the authors state that multiplexing approaches “identify only a fraction of the doublets” — this is technically true, but a bit misleading. The fraction of doublets detected in multiplexing experiments scales with the number of multiplexed samples. In instances where only a handful of samples are multiplexed, it is fair to say that ‘only a fraction' are identified. However, any modestly large experiment will result in the *vast* majority of doublets being detected. And even in the lowly multiplexed contexts, it is straightforward to remove heterotypic at the cluster-level by identifying clusters enriched for empirically-defined doublets. Authors should clarify (or remove) this statement as it suggests that scDblFinder is a ‘competitor’ with multiplexing approaches for doublet detection performance. Computational prediction will very rarely out-compete empirical measurements — instead, scDblFinder has the key advantage of being applicable to any past or future scRNA-seq data, which is not the case for Cell Hashing, MULTI-seq, etc.

Are the conclusions about the tool and its performance adequately supported by the findings presented in the article?

Yes

Is the rationale for developing the new software tool clearly explained?

Yes

Is the description of the software tool technically sound?

Yes

Are sufficient details of the code, methods and analysis (if applicable) provided to allow replication of the software development and its use by others?

Yes

Is sufficient information provided to allow interpretation of the expected output datasets and any results generated using the tool?

Yes

Reviewer Expertise:

Tissue engineering and single cell analysis method development

We confirm that we have read this submission and believe that we have an appropriate level of expertise to confirm that it is of an acceptable scientific standard, however we have significant reservations, as outlined above.

## References

[1] : Benchmarking Computational Doublet-Detection Methods for Single-Cell RNA Sequencing Data. *Cell Systems* .2021;12(2) : 10.1016/j.cels.2020.11.008 176-194.e6 10.1016/j.cels.2020.11.008 PMC789725033338399

[2] : Chord: Identifying Doublets in Single-Cell RNA Sequencing Data by an Ensemble Machine Learning Algorithm. *bioRxiv* .2021; 10.1101/2021.05.07.442884 10.1101/2021.05.07.442884

# Response

**Published:** 2022-04-23

**Authors:** Pierre-Luc Germain

We thank the reviewers for their thorough reading and useful comments, and have tried to address all of them in the revised version. A detailed point-by-point version will be made available along with the new version, but here are the main points of response:

1. **Artificial doublet generation: **The reviewers are right that we simply assumed, because the doublets showed lower library size than expected from summation, that the mixed strategy would be superior. We now tested this, along with averaging. While averaging expectedly resulted in poorer performance, we in fact did not see a clear overall improvement of the mixed strategy. We now discuss this explicitly.
2. **Thresholding:** Following the reviewers' suggestions, we also rewrote much of this section in a more open fashion, reflecting the choices that ultimately enter any thresholding decision (i.e. the lack of an objective optimal threshold), and changed the figure to provide clearer readouts that compare more alternative procedures.
3. **scATACseq:** Thanks for the suggestions, at the time of submission we were not aware of the AMULET method. We now included it in the comparison, along with the dataset with ground truth with which it was published. We also reimplemented the method in our package, for the convenience of R users.
4. **Text structure and clarity:** With permission from f1000, we now moved the Methods section to the end (as it was originally conceived), tried to offer a simpler overview of the method in the Results section, and to clarify the methods.
5. Finally we also addressed the minor comments.
